# Supplementary material for: Longitudinal employment patterns and parental health: A cross-country look
Source: PLoS One. 2026 Jun 5;21(6):e0350945. doi: 10.1371/journal.pone.0350945 (PMC13240889; doi:10.1371/journal.pone.0350945)
Supplement: S1 Table — (DOCX) [file pone.0350945.s001.docx]

**S1. Table. Measures and Variable Comparability**

| **Variables** | **Australia: HILDA** | **Germany: SOEP** | **UK: UKHLS** | **US: NLSY79** |
| --- | --- | --- | --- | --- |
| **HEALTH OUTCOMES** |  |  |  |  |
| Physical Health/Function  (Standardized score with a mean of 50 and a standard deviation of 10) | SF-36 Physical Health   - Vigorous activities (1-limited a lot, 2-limited a little, 3-not limited at all) - Moderate activities (1-3) - Lifting or carrying groceries (1-3) - Climbing several flights of stairs (1-3) - Climbing one flight of stairs (1-3) - Bending kneeling or stooping (1-3) - Walking more than one kilometer (1-3) - Walking half a kilometer (1-3) - Walking 100 meters (1-3) - Bathing or dressing yourself (1-3) | - State of health affects ascending stairs (1-strong; 2-a little; 3-not at all) - State of health affects tiring tasks (1-strong; 2-a little; 3-not at all) - Have trouble climbing stairs (y/n) - Dressing difficult alone (y/n) - Difficulty/need help getting in/out bed (y/n) - Need help with shopping (y/n)   Housework difficult alone (y/n) | [SF-12 Physical Component Summary](https://www.understandingsociety.ac.uk/documentation/mainstage/variables/sf12pcs_dv/)   - - General health (1 excellent – 5 poor) - - Health limits typical activities (1-no, 2- a little, 3- a lot) - - Health limits several flights of stairs (1-no, 2- a little, 3- a lot) - - Health limits work (1 yes – 0 no) - - Accomplished less (1 yes – 0 no) - - Pain interferes with work (1 yes – 0 no) | SF-12 Physical Component  Summary   - - General health (1 excellent – 5 poor) - - Health limits moderate activities (1-no, 2- a little, 3- a lot) - - Health limits climb stairs (1-no, 2-a little, 3-a lot) - Accomplish less than would like (1 yes – 0 no) - Health limits work (1 yes – 0 no) - Pain interferes with normal work (1 yes – 0 no) |
| Mental Health/Function  Standardized score with a mean of 50 and a standard deviation of 10) | SF-36 Physical Health   - Been a nervous person (1-6: 1-all of the time to 6-none of the time) - Felt so down in the dumps nothing could cheer you up (1-6) - Felt calm and peaceful (1-6) - Felt down (1-6) - Been a happy person (1-6) | - Run-down, melancholy (depressed/sad) in last 4 weeks (1-5: always/ often/ sometimes/ almost never/never) - Well-balanced in last 4 weeks (1-5: always/ often/ sometimes/ almost never/never) | SF-12 Mental Component Summary   - Accomplish less than would like (1 yes – 0 no) - Emotional problems cause to accomplish less (1 yes – 0 no) - Emotional problems made less careful (1 yes – 0 no) - Felt calm and peaceful (1 all the time – 6 none of the time) - Had a lot of energy (1 all the time – 6 none of the time) - Felt downhearted and blue (1 all the time – 6 none of the time)   Physical and emotional problems interfere with social activities (1 all the time – 3 good bit of time) | SF-12 Mental Component Summary   - Accomplish less than would like (1 yes – 0 no) - Emotional problems cause to accomplish less (1 yes – 0 no) - Emotional problems made less careful (1 yes – 0 no) - Felt calm and peaceful (1 all the time – 6 none of the time) - Had a lot of energy (1 all the time – 6 none of the time) - Felt downhearted and blue (1 all the time – 6 none of the time) - Physical and emotional problems interfere with social activities (1 all the time – 3 good bit of time) |
| Self-Assessed Subjective Well-Being | 1 = Fair or Poor  0 = Good, Very good, or Excellent | 1 = Fair or Poor  0 = Good, Very good, or Excellent | 1 = Fair or Poor  0 = Good, Very good, or Excellent | 1 = Fair or Poor  0 = Good, Very good, or Excellent |
|  |  |  |  |  |
| Depression / Anxiety | Kessler-10 Distress Scale Score: continuous or a dichotomous variable with a value of 1 if score>=25 and 0 otherwise [1,2] |  | GHQ-12: continuous or a dichotomous variable with a value of 1 if score>=13 and 0 otherwise [3] | CED-D depressive symptoms [4]: continuous variable or a dichotomous variable with a value of 1 if score>=8 and 0 otherwise |
|  |  |  |  |  |
| **WORK SCHEDULE** |  |  |  |  |
|  | - Not working [NW] - Regular daytime schedule [ST] - Regular evening shift - Regular night shift - Other Nonstandard schedules [Other NST] (e.g., rotating shift, split shift, irregular hours, on call) | - Not working [NW] - Regular daytime schedule [ST] - Regular evening shift (after 7-10 pm either daily or at least twice per week, or rotating shift) - Regular night shift (after 10 pm either daily or at least twice per week or rotating shift) - Other NST (i.e., work weekends at least every other week) | - Not working [NW] - Regular daytime schedule [ST] - Regular evening shift - Regular night shift - Other NST (e.g., rotating shift, split shift, irregular hours, on call, or work weekends most or every weekend) | - Not working [NW] - Regular daytime schedule [ST] - Regular evening shift - Regular night shift - Other NST (e.g., rotating shift, split shift, irregular hours) |
| **SOCIODEMOGRAPHIC CHARACTERISTICS** |  |  |  |  |
| Gender | 1= male 0 = female | 1= male 0 = female | 1= male 0 = female | 1= male 0 = female |
| Age | Years | Years | Years | Years |
| Race-Ethnicity | Non-Indigenous Australians (ref.)  Indigenous Australians  English speaking migrants  Non-English migrants | Native-born (ref)  Foreign-born | - White (ref.)  - Black  - Mixed  - Asian  - Other | Non-Hispanic White (ref.)  Non-Hispanic Black  Hispanic  Others (Asian, multi-races, etc.) |
| Education | - Low education level (11 years or below) - Medium education level (12 years or Cert III/IV) - High education level (Adv diploma; bachelor; grad diploma; postgrad; with tertiary education)   [1] Postgrad (master/doctor)  [2] Grad diploma/grad cert  [3] Bachelor/honours  [4] Adv diploma, diploma  [5] Cert III/IV  [6] Year 12  [7] Year 11 and below | - Low education level (ISCED-97: 0 to 2) - Medium education level (ISCED-97: 3 to 4) - High education level (ISCED-97: 5-6)   ISCED-97:  [1] primary education  [2] lower secondary  [3] Upper secondary  [4] post secondary non-tertiary  [5] first stage of tertiary  [6] second stage of tertiary | - Low education level (ISCED-97: 0 to 2) - Medium education level (ISCED-97: 3 to 4) - High education level (ISCED-97: 5-6)   ISCED-97:  [1] primary education  [2] lower secondary  [3] Upper secondary  [4] post secondary non-tertiary  [5] first stage of tertiary  [6] second stage of tertiary | - Low education level (less than high school) - Medium education level (high school degree and some college) - High education level (college degree and above) |
| Partnership | - Partnered (married or cohabited) (ref.) - Not-partnered (separated, divorced, widowed, never married) | - Partnered (married or cohabited) (ref.)   Not-partnered (separated, divorced, widowed, never married) | - Partnered (married or cohabited) (ref.)   Not-partnered (separated, divorced, widowed, never married) | - Partnered (married) (ref.)   Not-partnered (separated, divorced, widowed, never married) |
| Parenthood status | Yes (1) vs. No (0) | Yes (1) vs. No (0) | Yes (1) vs. No (0) | Yes (1) vs. No (0) |
| Occupation | - Professionals/Managers/Technicians and Associate Professionals - Clerical Support / Clerks / Service-related workers / Sales-related workers (ref.) - All others   1-digit ANZSCO 2006 (8 occupations)  [1] Managers  [2] Professionals  [3] Technicians and Trades Workers  [4] Community and Personal Service Work  [5] Clerical and Administrative Workers  [6] Sales Workers  [7] Machinery Operators and Drivers  [8] Labourers | - Professionals/Managers/Technicians and Associate Professionals - Clerical Support / Cherks / Service-related workers / Sales-relate workers (ref.) - All others   ISCO-88 categories:  [1] Legislators, senior officials and managers  [2] Professionals  [3] Technicians and associate professionals  [4] Clerks  [5] Service workers and shop and market sales workers  [6] Skilled agricultural and fishery workers  [7] Craft and related workers  [8] Plant and machine operators and assemblers  [9] Elementary occupations  [0] Armed forces (excluded) | - Professionals/Managers/Technicians and Associate Professionals - Clerical Support / Cherks / Service-related workers / Sales-relate workers (ref.) - All others   ISCO88 categories:  [1] Legislators, senior officials and managers  [2] Professionals  [3] Technicians and associate professionals  [4] Clerks  [5] Service workers and shop and market sales workers  [6] Skilled agricultural and fishery workers  [7] Craft and related workers  [8] Plant and machine operators and assemblers  [9] Elementary occupations  [0] Armed forces (excluded) | - Professionals/Managers/Technicians and Associate Professionals - Clerical Support/Cherks/Service-related workers/Sales-relate workers (ref.) - All others |
| Weekly working hours | - Average working hours between 22-34, 35-44, 45-54 - A dichotomous variable indicating Full-time (>=35 hours/wk) vs. Part-time (<35 hours/wk) | - Average working hours between 22-34, 35-44, 45-54 - A dichotomous variable indicating Full-time (>=35 hours/wk) vs. Part-time (<35 hours/wk) | - Average working hours between 22-34, 35-44, 45-54 - A dichotomous variable indicating Full-time (>=35 hours/wk) vs. Part-time (<35 hours/wk) | - Average working hours between 22-34, 35-44, 45-54 - A dichotomous variable indicating Full-time (>=35 hours/wk) vs. Part-time (<35 hours/wk) |

*Note*. ref: reference group in the multiple regression analysis.

1. Andrews G, Slade T. Interpreting scores on the Kessler Psychological Distress Scale (k10). Aust N Z J Public Health. 2001; 25: 494–7. <https://doi.org/10.1111/j.1467-842x.2001.tb00310.x>
2. Kessler RC, Andrews G, Colpe LJ, Hiripi E, Mroczek DK, Normand SLT, Walters EE, Zaslavsky AM. Short screening scales to monitor population prevalences and trends in non-specific psychological distress. Psychol Med. 2002; 32: 959-956. <https://doi.org/10.1017/s0033291702006074>
3. Goldberg DP, Hillier VF. A scaled version of the General Health Questionnaire. Psychol Med. 1979; 9(1): 139–45. <https://doi.org/10.1017/S0033291700021644>
4. Radloff LS. The CES-D Scale: A self-report depression scale for research in the general population. Appl Psychol Meas. 1977; 1(3): 385–401. https://doi.org/10.1177/014662167700100306
