# Supplementary material for: Longitudinal employment patterns and parental health: A cross-country look
Source: PLoS One. 2026 Jun 5;21(6):e0350945. doi: 10.1371/journal.pone.0350945 (PMC13240889; doi:10.1371/journal.pone.0350945)
Supplement: S2 Table — (DOCX) [file pone.0350945.s002.docx]

**S2. Table. Work Arrangement Sequence Cluster Solution Diagnostic Indices**

**Panel A. Parents Aged 25-34**

| Number of clusters | Calinski/Harabasz pseudo-F | Duda/Hart |  |
| --- | --- | --- | --- |
|  |  | Je(2)/Je(1) | Pseudo T-squared |
| *Australia HILDA (n = 2,781)* |  |  |  |
| 2 | 0.97 | 1.0000 | 0.06 |
| 3 | 0.52 | 0.9998 | 0.13 |
| **4** | **0.39** | **0.9999** | **0.06** |
| 5 | 0.30 | 0.9732 | 38.18 |
| 6 | 7.97 | 0.9939 | 3.37 |
| 7 | 7.20 | 0.9693 | 30.58 |
| 8 | 11.24 | 0.9998 | 0.08 |
| 9 | 9.84 | 0.9957 | 1.51 |
| 10 | 8.93 | 0.9643 | 25.38 |
| 11 | 10.67 | 0.9946 | 1.17 |
| 12 | 9.83 | 0.9893 | 3.14 |
| 13 | 9.29 | 0.9902 | 1.95 |
| 14 | 8.75 | 0.9894 | 0.45 |
| 15 | 8.17 | 0.9932 | 1.35 |
| *Germany SOEP (n = 1,165)* |  |  |  |
| 2 | 22.97 | 0.9857 | 9.36 |
| 3 | 16.57 | 0.9992 | 0.32 |
| **4** | **11.16** | **0.9996** | **0.09** |
| 5 | 8.39 | 0.9954 | 2.39 |
| 6 | 7.15 | 0.9995 | 0.16 |
| 7 | 5.98 | 0.9931 | 1.77 |
| 8 | 5.40 | 0.9934 | 1.22 |
| 9 | 4.88 | 0.9991 | 0.14 |
| 10 | 4.35 | 0.9938 | 0.99 |
| 11 | 4.00 | 0.9600 | 8.33 |
| 12 | 4.24 | 0.9717 | 2.48 |
| 13 | 4.11 | 0.9941 | 0.72 |
| 14 | 3.86 | 0.9996 | 0.05 |
| 15 | 3.58 | 0.9970 | 0.24 |
| *UK HLS (n = 1,771)* |  |  |  |
| 2 | 11.24 | 0.9816 | 15.03 |
| 3 | 13.29 | 0.9996 | 0.42 |
| **4** | **9.00** | **0.9988** | **0.42** |
| 5 | 6.85 | 0.9989 | 0.49 |
| 6 | 5.58 | 1.0000 | 0.00 |
| 7 | 4.65 | 0.9979 | 0.92 |
| 8 | 4.12 | 0.9999 | 0.05 |
| 9 | 3.61 | 0.9999 | 0.04 |
| 10 | 3.21 | 0.9970 | 0.80 |
| 11 | 2.97 | 0.9955 | 0.90 |
| 12 | 2.78 | 0.9938 | 1.78 |
| 13 | 2.70 | 0.9852 | 1.18 |
| 14 | 2.58 | 0.9890 | 1.48 |
| 15 | 2.51 | 0.9999 | 0.02 |
| *US NLSY79 (n = 6,497)* |  |  |  |
| 2 | 4.92 | 0.9994 | 2.00 |
| 3 | 3.46 | 0.9921 | 19.77 |
| **4** | **8.87** | **0.9998** | **0.41** |
| 5 | 6.75 | 0.9974 | 7.55 |
| 6 | 6.91 | 0.9911 | 9.60 |
| 7 | 7.39 | 0.9975 | 1.63 |
| 8 | 6.59 | 0.9986 | 1.37 |
| 9 | 5.94 | 0.9950 | 3.61 |
| 10 | 5.67 | 0.9987 | 1.09 |
| 11 | 5.20 | 0.9998 | 0.35 |
| 12 | 4.76 | 0.9983 | 0.91 |
| 13 | 4.43 | 0.9947 | 3.26 |
| 14 | 4.34 | 0.9999 | 0.02 |
| 15 | 4.03 | 0.9891 | 2.88 |

*Note*. Bolded cluster solution was chosen as the ideal number of cluster solutions based on the diagnostic indices.

**Panel B. Parents Aged 35-44**

| Number of clusters | Calinski/Harabasz pseudo-F | Duda/Hart |  |
| --- | --- | --- | --- |
|  |  | Je(2)/Je(1) | Pseudo T-squared |
| *Australia HILDA (n = 1,870)* |  |  |  |
| 2 | 0.00 | 1.0000 | 0.02 |
| 3 | 0.01 | 0.9984 | 0.88 |
| 4 | 0.30 | 0.9817 | 20.03 |
| **5** | **5.10** | **1.0000** | **0.02** |
| 6 | 4.08 | 0.9557 | 28.70 |
| 7 | 9.16 | 0.9994 | 0.17 |
| 8 | 7.87 | 0.9831 | 2.54 |
| 9 | 7.25 | 0.9929 | 1.76 |
| 10 | 6.66 | 0.9992 | 0.09 |
| 11 | 6.00 | 0.9996 | 0.18 |
| 12 | 5.47 | 0.9427 | 9.55 |
| 13 | 6.00 | 0.9975 | 0.35 |
| 14 | 5.59 | 0.9754 | 2.85 |
| 15 | 5.38 | 0.9992 | 0.12 |
| *Germany SOEP (n = 2,823)* |  |  |  |
| 2 | 0.28 | 0.9969 | 3.85 |
| 3 | 2.02 | 0.9982 | 1.74 |
| 4 | 1.91 | 0.9950 | 7.97 |
| **5** | **3.46** | **0.9998** | **0.13** |
| 6 | 2.79 | 0.9993 | 0.61 |
| 7 | 2.43 | 0.9996 | 0.19 |
| 8 | 2.11 | 0.9978 | 1.68 |
| 9 | 2.05 | 0.9999 | 0.04 |
| 10 | 1.84 | 0.9995 | 0.21 |
| 11 | 1.67 | 0.9986 | 0.42 |
| 12 | 1.55 | 0.9961 | 1.02 |
| 13 | 1.51 | 0.9964 | 1.19 |
| 14 | 1.48 | 0.9998 | 0.10 |
| 15 | 1.38 | 0.9953 | 1.21 |
| *UK HLS (n = 3,363)* |  |  |  |
| 2 | 9.91 | 0.9955 | 7.46 |
| 3 | 8.67 | 0.9993 | 0.63 |
| 4 | 5.99 | 0.9957 | 7.25 |
| **5** | **6.32** | **0.9998** | **0.13** |
| 6 | 5.08 | 0.9999 | 0.04 |
| 7 | 4.24 | 0.9983 | 1.01 |
| 8 | 3.78 | 0.9983 | 0.63 |
| 9 | 3.38 | 0.9976 | 2.26 |
| 10 | 3.25 | 0.9791 | 7.76 |
| 11 | 3.71 | 0.9999 | 0.02 |
| 12 | 3.37 | 0.9971 | 2.14 |
| 13 | 3.26 | 0.9985 | 0.64 |
| 14 | 3.06 | 0.9937 | 2.21 |
| 15 | 3.00 | 0.9954 | 1.55 |
| *US NLSY79 (n = 6,352)* |  |  |  |
| 2 | 4.44 | 0.9977 | 9.48 |
| 3 | 6.97 | 0.9962 | 12.56 |
| **4** | **8.80** | **0.9991** | **2.57** |
| 5 | 7.24 | 0.9981 | 3.34 |
| 6 | 6.47 | 0.9957 | 4.22 |
| 7 | 6.07 | 0.9987 | 1.58 |
| 8 | 5.44 | 0.9995 | 0.45 |
| 9 | 4.81 | 0.9982 | 0.99 |
| 10 | 4.38 | 0.9995 | 0.38 |
| 11 | 3.98 | 0.9996 | 0.15 |
| 12 | 3.63 | 0.9909 | 2.68 |
| 13 | 3.53 | 0.9996 | 0.17 |
| 14 | 3.27 | 0.9998 | 0.05 |
| 15 | 3.04 | 0.9905 | 4.21 |

*Note*. Bolded cluster solution was chosen as the ideal number of cluster solutions based on the diagnostic indices.

**Panel C. Parents Aged 45-54**

| Number of clusters | Calinski/Harabasz pseudo-F | Duda/Hart |  |
| --- | --- | --- | --- |
|  |  | Je(2)/Je(1) | Pseudo T-squared |
| *Australia HILDA (n = 2,429)* |  |  |  |
| 2 | 1.39 | 1.0000 | 0.00 |
| 3 | 0.69 | 0.9936 | 4.09 |
| 4 | 1.74 | 0.9824 | 27.49 |
| 5 | 8.44 | 0.9866 | 11.94 |
| 6 | 9.87 | 0.9974 | 1.13 |
| **7** | **8.43** | **0.9999** | **0.07** |
| 8 | 7.23 | 0.9926 | 1.42 |
| 9 | 6.50 | 0.9833 | 4.07 |
| 10 | 6.31 | 0.9206 | 23.73 |
| 11 | 8.35 | 0.9956 | 0.91 |
| 12 | 7.66 | 0.9937 | 0.86 |
| 13 | 7.08 | 0.9820 | 3.65 |
| 14 | 7.04 | 0.9965 | 0.88 |
| 15 | 6.60 | 0.9926 | 2.99 |
| *Germany SOEP (n = 2,673)* |  |  |  |
| 2 | 5.54 | 1.0000 | 0.0 |
| 3 | 2.77 | 0.9959 | 4.07 |
| 4 | 3.23 | 0.9897 | 6.46 |
| 5 | 4.00 | 0.9885 | 14.75 |
| **6** | **6.13** | **0.9987** | **0.53** |
| 7 | 5.20 | 0.9591 | 38.61 |
| 8 | 9.81 | 0.9751 | 9.28 |
| 9 | 9.87 | 0.9638 | 12.78 |
| 10 | 10.27 | 0.9995 | 0.12 |
| 11 | 9.25 | 0.9972 | 1.01 |
| 12 | 8.50 | 0.9936 | 2.87 |
| 13 | 8.06 | 0.9941 | 0.81 |
| 14 | 7.50 | 0.9959 | 0.96 |
| 15 | 7.04 | 0.9833 | 4.72 |
| *UK HLS (n = 2,671)* |  |  |  |
| 2 | 6.46 | 0.9996 | 0.65 |
| 3 | 3.56 | 0.9999 | 0.16 |
| **4** | **2.42** | **0.9999** | **0.14** |
| 5 | 1.85 | 0.9999 | 0.03 |
| 6 | 1.49 | 0.9973 | 2.23 |
| 7 | 1.63 | 0.9967 | 0.97 |
| 8 | 1.54 | 0.9982 | 0.29 |
| 9 | 1.38 | 0.9992 | 0.64 |
| 10 | 1.30 | 0.9971 | 1.54 |
| 11 | 1.33 | 0.9951 | 1.38 |
| 12 | 1.33 | 0.9968 | 1.60 |
| 13 | 1.35 | 0.9935 | 1.44 |
| 14 | 1.36 | 0.9821 | 5.77 |
| 15 | 1.69 | 1.0000 | 0.00 |
| *US NLSY79 (n = 4,919)* |  |  |  |
| 2 | 3.39 | 0.9997 | 0.85 |
| 3 | 2.11 | 0.9999 | 0.21 |
| 4 | 1.48 | 0.9965 | 7.33 |
| 5 | 2.91 | 0.9976 | 3.76 |
| 6 | 3.05 | 0.9967 | 4.00 |
| **7** | **3.18** | **0.9994** | **0.31** |
| 8 | 2.77 | 0.9970 | 2.64 |
| 9 | 2.74 | 0.9984 | 0.93 |
| 10 | 2.54 | 1.0000 | 0.00 |
| 11 | 2.28 | 0.9978 | 0.97 |
| 12 | 2.16 | 0.9984 | 0.80 |
| 13 | 2.04 | 0.9744 | 4.15 |
| 14 | 2.18 | 0.9945 | 1.88 |
| 15 | 2.16 | 0.9978 | 0.60 |

*Note*. Bolded cluster solution was chosen as the ideal number of cluster solutions based on the diagnostic indices.

Milligan GW and Cooper MC. An examination of procedures for determining the number of clusters in a data set. Psychometrika. 1985; 50(2): 159–179.
