# Supplementary material for: Longitudinal employment patterns and parental health: A cross-country look
Source: PLoS One. 2026 Jun 5;21(6):e0350945. doi: 10.1371/journal.pone.0350945 (PMC13240889; doi:10.1371/journal.pone.0350945)
Supplement: S3 Table — (DOCX) [file pone.0350945.s003.docx]

**S3. Table. Descriptive Statistics of Analyzed Variables by Country and Age**

|  | **By the schedule patterns between ages 25-34** | | | | | **Sig.** | **# missing cases** |
| --- | --- | --- | --- | --- | --- | --- | --- |
| **HILDA (Australia)** | **Total (n = 2781)** | **Mainly NW (n = 255, 9.17%)** | **NW+ST (n = 553, 19.88%)** | **Other NST+ ST (n = 582, 20.93%)** | **Mainly ST (n = 1391, 50.02%)** |  |  |
| **Gender (%)** |  |  |  |  |  | *** |  |
| Female | 56.31 | 86.27 | 80.65 | 47.59 | 44.79 |  |  |
| Male | 43.69 | 13.73 | 19.35 | 52.41 | 55.21 |  |  |
| **Migration background (%)** |  |  |  |  |  | *** | 1 |
| Non-Indigenous Australian | 81.80 | 74.12 | 78.48 | 85.05 | 83.17 |  |  |
| Indigenous/Torres Strait Islander Australian | 4.28 | 15.29 | 5.79 | 2.75 | 2.30 |  |  |
| Other English-speaking country | 5.04 | 3.14 | 4.70 | 4.47 | 5.76 |  |  |
| Non-English-speaking country | 8.88 | 7.45 | 11.03 | 7.73 | 8.78 |  |  |
| **Background at age 25** |  |  |  |  |  |  |  |
| **Education (%)** |  |  |  |  |  | *** | 19 |
| Low education level | 18.9 | 45.88 | 26.78 | 14.43 | 12.65 |  |  |
| Medium education level | 47.21 | 44.31 | 47.36 | 52.75 | 45.35 |  |  |
| High education level | 33.89 | 9.80 | 25.87 | 32.82 | 42.01 |  |  |
| **Relationship status (%)** |  |  |  |  |  | * | 18 |
| Not-partnered | 25.44 | 32.55 | 26.96 | 26.12 | 23.24 |  |  |
| Partnered | 74.56 | 67.45 | 73.04 | 73.88 | 76.76 |  |  |
| **Parenthood status (%)** |  |  |  |  |  | *** |  |
| No | 58.76 | 23.14 | 45.39 | 64.26 | 68.30 |  |  |
| Yes | 41.24 | 76.86 | 54.61 | 35.74 | 31.70 |  |  |
| **Work variables between ages 25-34** |  |  |  |  |  |  |  |
| **Occupation (%)** |  |  |  |  |  | *** |  |
| Occupation missing (primarily due to not working) | 6.15 | 61.57 | 2.53 | 0.00 | 0.00 |  | 171 |
| Other occupations | 21.29 | 20.39 | 30.56 | 22.16 | 17.40 |  |  |
| Clerks/Service- and Sales-related Workers | 23.23 | 14.12 | 36.89 | 22.51 | 19.77 |  |  |
| Professionals/Managers/Technicians and Associate Professionals | 49.33 | 3.92 | 30.02 | 55.33 | 62.83 |  |  |
| **Weekly working hours (%)** |  |  |  |  |  | *** |  |
| Weekly hours missing (primarily due to not working) | 6.15 | 61.57 | 2.53 | 0.00 | 0.00 |  |  |
| Equal share of part- and full-time | 3.45 | 4.71 | 5.24 | 2.75 | 2.80 |  |  |
| Part-time (1-34 hrs/wk) | 26.03 | 25.88 | 51.36 | 27.15 | 15.53 |  |  |
| Full-time (> = 35 hrs/wk) | 64.37 | 7.84 | 40.87 | 70.10 | 81.67 |  |  |
| Average weekly working hours (mean) | 29.48 (n = 2781) | 1.49 (n = 255) | 14.97 (n = 553) | 36.74 (n = 582) | 37.35 (n = 1391) | *** |  |
| **Health variables at age 35** |  |  |  |  |  |  |  |
| SF-36 General health (0-100) | 49.19 (n = 1895) | 44.86 (n = 155) | 47.97 (n = 391) | 50.00 (n = 375) | 50.05 (n = 974) | *** | 886 |
| SF-36 Mental health (0-100) | 49.40 (n = 1901) | 45.00 (n = 158) | 47.50 (n = 392) | 50.15 (n = 375) | 50.59 (n = 976) | *** | 880 |
| Self-assessed general poor/fair health | 9.82 | 23.57 | 14.87 | 7.20 | 6.58 | *** | 886 |
| Kessler score (10-50) | 16.41 (n = 1940) | 20.60 (n = 163) | 17.88 (n = 400) | 15.62 (n = 377) | 15.64 (n = 1000) | *** | 841 |
| Kessler scale: risk of psychological distress (>=25) | 13.25 | 35.58 | 18.25 | 8.49 | 9.40 | *** | 841 |

*Note*. NW: not working; ST: standard daytime hours; other NST: non-daytime hours other than evenings/nights (e.g., weekends, irregular). Bivariate statistical significant tests were done by Chi-square test for categorical variables or ANOVA for continuous variables. * *p* < .05, ** *p* < .01, *** *p* < .001.

|  | **By the schedule patterns between ages 35-44** | | | | | | **Sig.** | **# missing cases** |
| --- | --- | --- | --- | --- | --- | --- | --- | --- |
| **HILDA (Australia)** | **Total (n = 1870)** | **Mainly NW (n = 249, 13.32%)** | **Volatile to Mainly ST (n = 411, 21.98%)** | **Mainly other NST (n = 133, 7.11%)** | **Mainly ST (n = 621, 33.21%)** | **ST only (n = 456, 24.39%)** |  |  |
| **Gender (%)** |  |  |  |  |  |  | *** |  |
| Female | 59.14 | 85.14 | 66.67 | 46.62 | 57.00 | 44.74 |  |  |
| Male | 40.86 | 14.86 | 33.33 | 53.38 | 43.00 | 55.26 |  |  |
| **Migration background (%)** |  |  |  |  |  |  | *** |  |
| Non-Indigenous Australian | 79.79 | 74.30 | 77.37 | 82.71 | 81.00 | 82.46 |  |  |
| Indigenous/Torres Strait Islander Australian | 2.46 | 6.83 | 1.95 | 1.50 | 2.25 | 1.10 |  |  |
| Other English-speaking country | 6.58 | 3.61 | 7.54 | 6.02 | 6.12 | 8.11 |  |  |
| Non-English-speaking country | 11.18 | 15.26 | 13.14 | 9.77 | 10.63 | 8.33 |  |  |
| **Background at age 25** |  |  |  |  |  |  |  |  |
| **Education (%)** |  |  |  |  |  |  | *** | 3 |
| Low education level | 20.51 | 41.37 | 24.63 | 18.05 | 14.86 | 13.82 |  |  |
| Medium education level | 38.35 | 34.54 | 40.00 | 44.36 | 40.23 | 34.65 |  |  |
| High education level | 41.14 | 24.10 | 35.37 | 37.59 | 44.91 | 51.54 |  |  |
| **Relationship status (%)** |  |  |  |  |  |  | *** | 3 |
| Not-partnered | 11.78 | 22.89 | 13.17 | 6.77 | 10.18 | 8.11 |  |  |
| Partnered | 88.22 | 77.11 | 86.83 | 93.23 | 89.82 | 91.89 |  |  |
| **Parenthood status (%)** |  |  |  |  |  |  |  | 26 |
| No | 3.09 | 2.88 | 4.23 | 2.27 | 3.08 | 2.44 |  |  |
| Yes | 96.91 | 97.12 | 95.77 | 97.73 | 96.92 | 97.56 |  |  |
| **Work variables between ages 25-34** |  |  |  |  |  |  |  |  |
| **Occupation (%)** |  |  |  |  |  |  | *** |  |
| Occupation missing (primarily due to not working) | 6.68 | 49.80 | 0.24 | 0.00 | 0.00 | 0.00 |  | 125 |
| Other occupations | 19.47 | 20.88 | 28.71 | 24.81 | 15.78 | 13.82 |  |  |
| Clerks/Service- and Sales-related Workers | 24.87 | 19.68 | 28.95 | 33.83 | 24.80 | 21.49 |  |  |
| Professionals/Managers/Technicians and Associate Professionals | 48.98 | 9.64 | 42.09 | 41.35 | 59.42 | 64.69 |  |  |
| **Weekly working hours (%)** |  |  |  |  |  |  | *** |  |
| Weekly hours missing (primarily due to not working) | 6.68 | 49.80 | 0.24 | 0.00 | 0.00 | 0.00 |  |  |
| Equal share of part- and full-time | 2.78 | 3.21 | 4.62 | 1.50 | 2.74 | 1.32 |  |  |
| Part-time (1-34 hrs/wk) | 33.16 | 40.16 | 47.69 | 31.58 | 32.21 | 17.98 |  |  |
| Full-time (> = 35 hrs/wk) | 57.38 | 6.83 | 47.45 | 66.92 | 65.06 | 80.70 |  |  |
| Average weekly working hours (mean) | 30.34 (n = 1870) | 2.64 (n = 249) | 26.82 (n = 411) | 37.48 (n = 133) | 35.07 (n = 621) | 40.09 (n = 456) | *** |  |
| **Health variables at age 35** |  |  |  |  |  |  |  |  |
| SF-36 General health (0-100) | 49.05 (n = 1222) | 43.41 (n = 164) | 49.03 (n = 255) | 48.58 (n = 93) | 49.96 (n = 322) | 50.80 (n = 388) | *** | 648 |
| SF-36 Mental health (0-100) | 49.78 (n = 1225) | 45.13 (n = 166) | 49.42 (n = 256) | 50.57 (n = 93) | 50.18 (n = 322) | 51.47 (n = 388) | *** | 645 |
| Self-assessed general poor/fair health | 14.06 | 33.13 | 14.17 | 13.04 | 11.80 | 7.97 | *** | 647 |
| Kessler score (10-50) | 16.19 (n = 1210) | 20.04 (n = 164) | 16.44 (n = 257) | 15.97 (n = 91) | 15.84 (n = 320) | 14.71 (n = 378) | *** | 660 |
| Kessler scale: risk of psychological distress (>=25) | 11.49 | 28.66 | 13.62 | 10.99 | 9.06 | 4.76 | *** | 660 |

*Note*. NW: not working; ST: standard daytime hours; other NST: non-daytime hours other than evenings/nights (e.g., weekends, irregular). Bivariate statistical significant tests were done by Chi-square test for categorical variables or ANOVA for continuous variables. * *p* < .05, ** *p* < .01, *** *p* < .001.

|  | **By the schedule patterns between ages 45-54** | | | | | | | | **Sig.** | **# missing cases** |
| --- | --- | --- | --- | --- | --- | --- | --- | --- | --- | --- |
| **HILDA (Australia)** | **Total (n = 2429)** | **Mainly NW (n = 250, 10.29%)** | **Volatile (n = 192, 7.90%)** | **ST+other NST (n = 241, 9.92%)** | **Mainly other NST (n = 208, 8.56%)** | **Mainly ST (n = 678, 27.91%)** | **Mainly ST to Missing (n = 201, 8.28%)** | **ST only (n = 659, 27.13%)** |  |  |
| **Gender (%)** |  |  |  |  |  |  |  |  | *** |  |
| Female | 49.81 | 79.60 | 59.38 | 44.81 | 41.83 | 49.26 | 39.80 | 43.70 |  |  |
| Male | 50.19 | 20.40 | 40.62 | 55.19 | 58.17 | 50.74 | 60.20 | 56.30 |  |  |
| **Migration background (%)** |  |  |  |  |  |  |  |  | *** |  |
| Non-Indigenous Australian | 72.70 | 61.60 | 65.10 | 69.71 | 76.44 | 74.34 | 70.65 | 78.00 |  |  |
| Indigenous/Torres Strait Islander Australian | 2.18 | 7.60 | 3.65 | 0.83 | 1.92 | 1.62 | 1.99 | 0.91 |  |  |
| Other English-speaking country | 11.16 | 6.40 | 13.02 | 15.35 | 11.06 | 11.50 | 12.94 | 10.02 |  |  |
| Non-English-speaking country | 13.96 | 24.40 | 18.23 | 14.11 | 10.58 | 12.54 | 14.43 | 11.08 |  |  |
| **Background at age 25** |  |  |  |  |  |  |  |  |  |  |
| **Education (%)** |  |  |  |  |  |  |  |  | *** | 6 |
| Low education level | 24.52 | 48.19 | 35.08 | 21.99 | 21.15 | 21.81 | 18.91 | 18.97 |  |  |
| Medium education level | 33.59 | 32.53 | 36.13 | 38.59 | 41.83 | 32.49 | 33.83 | 29.89 |  |  |
| High education level | 41.89 | 19.28 | 28.80 | 39.42 | 37.02 | 45.70 | 47.26 | 51.14 |  |  |
| **Relationship status (%)** |  |  |  |  |  |  |  |  | *** | 6 |
| Not-partnered | 14.86 | 27.31 | 19.37 | 14.11 | 12.98 | 13.35 | 10.95 | 12.44 |  |  |
| Partnered | 85.14 | 72.69 | 80.63 | 85.89 | 87.02 | 86.65 | 89.05 | 87.56 |  |  |
| **Parenthood status (%)** |  |  |  |  |  |  |  |  |  |  |
| No | 8.19 | 10.00 | 11.98 | 7.05 | 9.62 | 7.82 | 8.46 | 6.68 |  |  |
| Yes | 91.81 | 90.00 | 88.02 | 92.95 | 90.38 | 92.18 | 91.54 | 93.32 |  |  |
| **Work variables between ages 25-34** |  |  |  |  |  |  |  |  |  |  |
| **Occupation (%)** |  |  |  |  |  |  |  |  | *** |  |
| Occupation missing (primarily due to not working) | 5.97 | 58.00 | 0.00 | 0.00 | 0.00 | 0.00 | 0.00 | 0.00 |  | 145 |
| Other occupations | 17.91 | 16.00 | 33.85 | 24.07 | 21.63 | 18.14 | 16.42 | 10.77 |  |  |
| Clerks/Service- and Sales-related Workers | 23.80 | 17.60 | 35.42 | 21.99 | 31.73 | 24.04 | 16.42 | 22.91 |  |  |
| Professionals/Managers/Technicians and Associate Professionals | 52.33 | 8.40 | 30.73 | 53.94 | 46.63 | 57.82 | 67.16 | 66.31 |  |  |
| **Weekly working hours (%)** |  |  |  |  |  |  |  |  | *** |  |
| Weekly hours missing (primarily due to not working) | 5.97 | 58.00 | 0.00 | 0.00 | 0.00 | 0.00 | 0.00 | 0.00 |  |  |
| Equal share of part- and full-time | 2.35 | 2.00 | 4.17 | 3.32 | 2.40 | 2.06 | 1.00 | 2.28 |  |  |
| Part-time (1-34 hrs/wk) | 27.09 | 34.00 | 50.00 | 30.71 | 33.65 | 25.22 | 23.88 | 17.30 |  |  |
| Full-time (> = 35 hrs/wk) | 64.59 | 6.00 | 45.83 | 65.98 | 63.94 | 72.71 | 75.12 | 80.42 |  |  |
| Average weekly working hours (mean) | 33.26 (n = 2429) | 1.74 (n = 250) | 19.92 (n = 192) | 38.73 (n = 241) | 37.73 (n = 208) | 36.83 (n = 678) | 37.55 (n = 201) | 40.71 (n = 659) | *** |  |
| **Health variables at age 35** |  |  |  |  |  |  |  |  |  |  |
| SF-36 General health (0-100) | 48.98 (n = 1702) | 43.64 (n = 171) | 43.94 (n = 133) | 50.00 (n = 180) | 48.2 (n = 158) | 49.47 (n = 485) | 47.94 (n = 21) | 51.34 (n = 554) | *** | 727 |
| SF-36 Mental health (0-100) | 49.80 (n = 1705) | 44.98 (n = 171) | 47.28 (n = 134) | 50.1 (n = 181) | 50.53 (n = 158) | 50.05 (n = 485) | 49.78 (n = 21) | 51.37 (n = 555) | *** | 724 |
| Self-assessed general poor/fair health | 18.40 | 34.32 | 37.88 | 16.11 | 19.50 | 16.08 | 23.81 | 11.17 | *** | 728 |
| Kessler score (10-50) | 15.60 (n = 1719) | 19.42  (n = 175) | 17.44 (n = 133) | 15.51 (n = 179) | 15.23 (n = 159) | 15.16 (n = 491) | 16.29 (n = 21) | 14.47 (n = 561) | *** | 710 |
| Kessler scale: risk of psychological distress (>=25) | 9.89 | 26.86 | 14.29 | 8.94 | 8.18 | 8.15 | 14.29 | 5.70 | *** | 710 |

*Note*. NW: not working; ST: standard daytime hours; other NST: non-daytime hours other than evenings/nights (e.g., weekends, irregular). Bivariate statistical significant tests were done by Chi-square test for categorical variables or ANOVA for continuous variables. * *p* < .05, ** *p* < .01, *** *p* < .001.

|  | **By the schedule patterns between ages 25-34** | | | | | **Sig.** | **# missing cases** |
| --- | --- | --- | --- | --- | --- | --- | --- |
| **SOEP (Germany)** | **Total (n = 1165)** | **Mainly NW to some ST+NST (n = 258, 22.15%)** | **Volatile (n = 204, 17.51%)** | **Volatile to Mainly Evenings (n = 184, 15.79%)** | **Mainly ST (n = 519, 44.55%)** |  |  |
| **Gender (%)** |  |  |  |  |  | *** |  |
| Female | 68.84 | 85.66 | 52.45 | 67.39 | 67.44 |  |  |
| Male | 31.16 | 14.34 | 47.55 | 32.61 | 32.56 |  |  |
| **Migration background (%)** |  |  |  |  |  |  |  |
| No migration background | 78.71 | 74.42 | 75.49 | 80.98 | 81.31 |  |  |
| First generation | 13.30 | 18.6 | 13.24 | 11.96 | 11.18 |  |  |
| Second generation | 7.98 | 6.98 | 11.27 | 7.07 | 7.51 |  |  |
| **Background at age 25** |  |  |  |  |  |  |  |
| **Education (%)** |  |  |  |  |  | *** | 198 |
| Low education level | 16.65 | 37.44 | 13.37 | 12.80 | 9.05 |  |  |
| Medium education level | 68.05 | 54.03 | 75.00 | 68.29 | 72.14 |  |  |
| High education level | 15.31 | 8.53 | 11.63 | 18.90 | 18.81 |  |  |
| **Relationship status (%)** |  |  |  |  |  |  | 176 |
| Not-partnered | 37.41 | 35.45 | 41.86 | 34.13 | 37.91 |  |  |
| Partnered | 62.59 | 64.55 | 58.14 | 65.87 | 62.09 |  |  |
| **Parenthood status (%)** |  |  |  |  |  | *** | 176 |
| No | 48.33 | 25.45 | 46.51 | 47.31 | 61.16 |  |  |
| Yes | 51.67 | 74.55 | 53.49 | 52.69 | 38.84 |  |  |
| **Work variables between ages 25-34** |  |  |  |  |  |  |  |
| **Occupation (%)** |  |  |  |  |  | *** |  |
| Occupation missing (primarily due to not working) | 8.15 | 33.72 | 0.49 | 0.00 | 1.35 |  | 95 |
| Other occupations | 21.20 | 16.28 | 36.27 | 13.04 | 20.62 |  |  |
| Clerks/Service- and Sales-related Workers | 25.49 | 26.36 | 21.57 | 29.35 | 25.24 |  |  |
| Professionals/Managers/Technicians and Associate Professionals | 45.15 | 23.64 | 41.67 | 57.61 | 52.79 |  |  |
| **Weekly working hours (%)** |  |  |  |  |  | *** |  |
| Weekly hours missing (primarily due to not working) | 5.67 | 25.58 | 0.00 | 0.00 | 0.00 |  |  |
| Equal share of part- and full-time | 5.41 | 6.20 | 5.39 | 4.35 | 5.39 |  |  |
| Part-time (1-34 hrs/wk) | 31.42 | 42.64 | 24.02 | 29.89 | 29.29 |  |  |
| Full-time (> = 35 hrs/wk) | 57.51 | 25.58 | 70.59 | 65.76 | 65.32 |  |  |
| Average weekly working hours (mean) | 24.28 (n = 1165) | 7.65 (n = 258) | 31.45 (n = 204) | 29.57 (n = 184) | 27.84 (n = 519) | *** |  |
| **Health variables at age 35** |  |  |  |  |  |  |  |
| **Short Form Health Survey (SF-12)** |  |  |  |  |  |  |  |
| Physical function (0-100) | 49.05 (n = 849) | 46.95 (n = 184) | 50.24 (n = 144) | 48.20 (n = 141) | 49.94 (n = 380) | ** | 316 |
| Mental function (0-100) | 50.11 (n = 849) | 48.52 (n = 184) | 51.85 (n = 144) | 49.72 (n = 141) | 50.37 (n = 380) | * | 316 |
| **Self-assessed general poor/fair health** | 11.71 | 19.11 | 10.67 | 10.37 | 8.95 | ** | 238 |

*Note*. NW: not working; ST: standard daytime hours; other NST: non-daytime hours other than evenings/nights (e.g., weekends, irregular). Bivariate statistical significant tests were done by Chi-square test for categorical variables or ANOVA for continuous variables. * *p* < .05, ** *p* < .01, *** *p* < .001.

|  | **By the schedule patterns between ages 35-44** | | | | | | **Sig.** | **# missing cases** |
| --- | --- | --- | --- | --- | --- | --- | --- | --- |
| **SOEP (Germany)** | **Total (n = 2823)** | **Mainly NW (n = 234, 8.29%)** | **Volatile (n = 678, 24.02%)** | **Mainly Nights (n = 310, 10.98%)** | **Volatile to mainly ST (n = 854, 30.25%)** | **Mainly ST (n = 747, 26.46%)** |  |  |
| **Gender (%)** |  |  |  |  |  |  | *** |  |
| Female | 60.61 | 87.18 | 56.05 | 40.97 | 67.33 | 56.89 |  |  |
| Male | 39.39 | 12.82 | 43.95 | 59.03 | 32.67 | 43.11 |  |  |
| **Migration background (%)** |  |  |  |  |  |  | *** |  |
| No migration background | 80.91 | 70.94 | 81.56 | 77.1 | 78.81 | 87.42 |  |  |
| First generation | 13.32 | 23.50 | 12.83 | 16.13 | 14.52 | 8.03 |  |  |
| Second generation | 5.77 | 5.56 | 5.6 | 6.77 | 6.67 | 4.55 |  |  |
| **Background at age 25** |  |  |  |  |  |  |  |  |
| **Education (%)** |  |  |  |  |  |  | *** | 4 |
| Low education level | 6.81 | 21.89 | 7.25 | 7.44 | 5.85 | 2.54 |  |  |
| Medium education level | 59.03 | 64.81 | 60.21 | 63.75 | 58.67 | 54.62 |  |  |
| High education level | 34.16 | 13.3 | 32.54 | 28.8 | 35.48 | 42.84 |  |  |
| **Relationship status (%)** |  |  |  |  |  |  |  |  |
| Not-partnered | 11.65 | 17.09 | 11.65 | 9.68 | 11.94 | 10.44 |  |  |
| Partnered | 88.35 | 82.91 | 88.35 | 90.32 | 88.06 | 89.56 |  |  |
| **Parenthood status (%)** |  |  |  |  |  |  | *** |  |
| No | 9.85 | 2.99 | 9.44 | 7.74 | 11.24 | 11.65 |  |  |
| Yes | 90.15 | 97.01 | 90.56 | 92.26 | 88.76 | 88.35 |  |  |
| **Work variables between ages 25-34** |  |  |  |  |  |  |  |  |
| **Occupation (%)** |  |  |  |  |  |  | *** |  |
| Occupation missing (primarily due to not working) | 5.84 | 60.26 | 0.29 | 0.65 | 1.76 | 0.67 |  | 165 |
| Other occupations | 21.93 | 17.95 | 22.12 | 38.39 | 23.42 | 14.46 |  |  |
| Clerks/Service- and Sales-related Workers | 23.06 | 13.68 | 27.14 | 14.84 | 24.59 | 23.96 |  |  |
| Professionals/Managers/Technicians and Associate Professionals | 49.17 | 8.12 | 50.44 | 46.13 | 50.23 | 60.91 |  |  |
| **Weekly working hours (%)** |  |  |  |  |  |  | *** |  |
| Weekly hours missing (primarily due to not working) | 4.46 | 53.42 | 0.00 | 0.00 | 0.12 | 0.00 |  |  |
| Equal share of part- and full-time | 2.02 | 0.00 | 1.92 | 1.94 | 3.28 | 1.34 |  |  |
| Part-time (1-34 hrs/wk) | 39.89 | 35.04 | 36.28 | 21.94 | 50.82 | 39.63 |  |  |
| Full-time (> = 35 hrs/wk) | 53.63 | 11.54 | 61.80 | 76.13 | 45.78 | 59.04 |  |  |
| Average weekly working hours (mean) | 29.12 (n = 2823) | 2.4 (n = 234) | 34.33 (n = 678) | 37.21 (n = 310) | 26.12 (n = 854) | 32.85 (n = 747) | *** |  |
| **Health variables at age 35** |  |  |  |  |  |  |  |  |
| **Short Form Health Survey (SF-12)** |  |  |  |  |  |  |  |  |
| Physical function (0-100) | 48.89 (n = 1895) | 45.34 (n = 161) | 48.77 (n = 473) | 49.23 (n = 203) | 48.85 (n = 569) | 50.07 (n = 489) | *** | 928 |
| Mental function (0-100) | 50.01 (n = 1895) | 47.39 (n = 161) | 50.26 (n = 473) | 50.22 (n = 203) | 49.76 (n = 569) | 50.84 (n = 489) | ** | 928 |
| **Self-assessed general poor/fair health** | 12.94 | 26.55 | 12.02 | 10.67 | 14.04 | 9.12 | *** | 690 |

*Note*. NW: not working; ST: standard daytime hours; other NST: non-daytime hours other than evenings/nights (e.g., weekends, irregular). Bivariate statistical significant tests were done by Chi-square test for categorical variables or ANOVA for continuous variables. * *p* < .05, ** *p* < .01, *** *p* < .001.

|  | **By the schedule patterns between ages 45-54** | | | | | | | **Sig.** | **# missing cases** |
| --- | --- | --- | --- | --- | --- | --- | --- | --- | --- |
| **SOEP (Germany)** | **Total (n = 2673)** | **Mainly NW to some ST (n = 421, 15.75%)** | **Mainly Nights (n = 342, 12.79%)** | **Mainly Evenings (n = 280, 10.48%)** | **Mainly other NST (n = 366, 13.69%)** | **Mainly ST to ST+NST (n = 356, 13.32%)** | **Mainly ST (n = 908, 33.97%)** |  |  |
| **Gender (%)** |  |  |  |  |  |  |  | *** |  |
| Female | 53.12 | 78.86 | 38.01 | 42.14 | 49.45 | 47.75 | 53.85 |  |  |
| Male | 46.88 | 21.14 | 61.99 | 57.86 | 50.55 | 52.25 | 46.15 |  |  |
| **Migration background (%)** |  |  |  |  |  |  |  | ** |  |
| No migration background | 89.19 | 85.27 | 86.26 | 87.14 | 91.53 | 89.61 | 91.63 |  |  |
| First generation | 8.64 | 12.35 | 11.4 | 9.29 | 7.65 | 7.02 | 6.72 |  |  |
| Second generation | 2.17 | 2.38 | 2.34 | 3.57 | 0.82 | 3.37 | 1.65 |  |  |
| **Background at age 25** |  |  |  |  |  |  |  |  |  |
| **Education (%)** |  |  |  |  |  |  |  | *** | 2 |
| Low education level | 5.35 | 14.96 | 5.85 | 3.23 | 3.01 | 3.37 | 3.08 |  |  |
| Medium education level | 54.96 | 62.23 | 56.14 | 46.24 | 57.53 | 44.66 | 56.83 |  |  |
| High education level | 39.69 | 22.80 | 38.01 | 50.54 | 39.45 | 51.97 | 40.09 |  |  |
| **Relationship status (%)** |  |  |  |  |  |  |  | *** |  |
| Not-partnered | 16.84 | 24.23 | 12.87 | 13.57 | 13.93 | 17.98 | 16.63 |  |  |
| Partnered | 83.16 | 75.77 | 87.13 | 86.43 | 86.07 | 82.02 | 83.37 |  |  |
| **Parenthood status (%)** |  |  |  |  |  |  |  |  |  |
| No | 2.88 | 2.85 | 0.88 | 2.5 | 3.83 | 4.49 | 2.75 |  |  |
| Yes | 97.12 | 97.15 | 99.12 | 97.5 | 96.17 | 95.51 | 97.25 |  |  |
| **Work variables between ages 25-34** |  |  |  |  |  |  |  |  |  |
| **Occupation (%)** |  |  |  |  |  |  |  | *** |  |
| Occupation missing (primarily due to not working) | 6.02 | 37.05 | 0.29 | 0.00 | 0.27 | 0.00 | 0.33 |  | 161 |
| Other occupations | 22.63 | 20.67 | 34.21 | 16.07 | 27.87 | 20.79 | 19.82 |  |  |
| Clerks/Service- and Sales-related Workers | 19.49 | 21.14 | 14.62 | 17.86 | 22.68 | 16.29 | 21.04 |  |  |
| Professionals/Managers/Technicians and Associate Professionals | 51.85 | 21.14 | 50.88 | 66.07 | 49.18 | 62.92 | 58.81 |  |  |
| **Weekly working hours (%)** |  |  |  |  |  |  |  | *** |  |
| Weekly hours missing (primarily due to not working) | 4.90 | 31.12 | 0.00 | 0.00 | 0.00 | 0.00 | 0.00 |  |  |
| Equal share of part- and full-time | 1.05 | 0.48 | 1.46 | 4.29 | 1.37 | 0.84 | 0.11 |  |  |
| Part-time (1-34 hrs/wk) | 88.25 | 67.46 | 83.63 | 76.43 | 90.98 | 96.35 | 99.01 |  |  |
| Full-time (> = 35 hrs/wk) | 5.80 | 0.95 | 14.91 | 19.29 | 7.65 | 2.81 | 0.88 |  |  |
| Average weekly working hours (mean) | 32.14 (n = 2673) | 7.78 (n = 421) | 39.73 (n = 342) | 41.91 (n = 280) | 37.22 (n = 366) | 36.54 (n = 356) | 33.80 (n = 908) | *** |  |
| **Health variables at age 35** |  |  |  |  |  |  |  |  |  |
| **Short Form Health Survey (SF-12)** |  |  |  |  |  |  |  |  |  |
| Physical function (0-100) | 48.14 (n = 1508) | 42.60 (n = 249) | 46.78 (n = 176) | 49.05 (n = 162) | 48.90 (n = 193) | 49.77 (n = 211) | 50.02 (n = 517) | *** | 1165 |
| Mental function (0-100) | 50.16 (n = 1508) | 48.56 (n = 249) | 50.18 (n = 176) | 50.20 (n = 162) | 51.54 (n = 193) | 50.72 (n = 211) | 50.17 (n = 517) |  | 1165 |
| **Self-assessed general poor/fair health** | 17.86 | 32.78 | 20.93 | 13.00 | 13.97 | 15.04 | 13.50 | *** | 881 |

*Note*. NW: not working; ST: standard daytime hours; other NST: non-daytime hours other than evenings/nights (e.g., weekends, irregular). Bivariate statistical significant tests were done by Chi-square test for categorical variables or ANOVA for continuous variables. * *p* < .05, ** *p* < .01, *** *p* < .001.

|  | **By the schedule patterns between ages 25-34** | | | | | **Sig.** | **# missing cases** |
| --- | --- | --- | --- | --- | --- | --- | --- |
| **UKHLS (United Kingdom)** | **Total (n = 1771)** | **Mainly NW to some ST+NST (n = 453, 25.58%)** | **Volatile to mainly other NST to Volatile (n = 351, 19.82%)** | **Mainly ST+ some NST (n = 554, 31.28%)** | **Mainly ST (n = 413, 23.32%)** |  |  |
| **Gender (%)** |  |  |  |  |  | *** |  |
| Female | 67.19 | 86.98 | 52.71 | 65.52 | 60.05 |  |  |
| Male | 32.81 | 13.02 | 47.29 | 34.48 | 39.95 |  |  |
| **Ethnicity (%)** |  |  |  |  |  | *** | 3 |
| White | 82.69 | 68.21 | 89.17 | 85.51 | 89.32 |  |  |
| Mixed | 1.81 | 2.65 | 1.14 | 1.63 | 1.70 |  |  |
| Asian | 12.16 | 23.18 | 7.69 | 10.33 | 6.31 |  |  |
| Black | 2.38 | 3.97 | 1.42 | 1.81 | 2.18 |  |  |
| Other | 0.96 | 1.99 | 0.57 | 0.72 | 0.49 |  |  |
| **Background at age 25** |  |  |  |  |  |  |  |
| **Education (%)** |  |  |  |  |  | *** | 11 |
| Low education level | 3.41 | 9.95 | 0.57 | 2.35 | 0.24 |  |  |
| Medium education level | 51.82 | 66.74 | 59.83 | 48.19 | 33.90 |  |  |
| High education level | 44.77 | 23.30 | 39.60 | 49.46 | 65.86 |  |  |
| **Relationship status (%)** |  |  |  |  |  | ** |  |
| Not-partnered | 28.12 | 33.11 | 24.79 | 29.96 | 23.00 |  |  |
| Partnered | 71.88 | 66.89 | 75.21 | 70.04 | 77.00 |  |  |
| **Parenthood status (%)** |  |  |  |  |  | *** |  |
| No | 50.76 | 21.85 | 51.28 | 58.66 | 71.43 |  |  |
| Yes | 49.24 | 78.15 | 48.72 | 41.34 | 28.57 |  |  |
| **Work variables between ages 25-34** |  |  |  |  |  |  |  |
| **Occupation (%)** |  |  |  |  |  | *** |  |
| Occupation missing (primarily due to not working) | 10.33 | 39.74 | 0.28 | 0.36 | 0.00 |  | 183 |
| Other occupations | 16.04 | 11.48 | 24.50 | 19.68 | 8.96 |  |  |
| Clerks/Service- and Sales-related Workers | 34.33 | 36.87 | 38.46 | 34.12 | 28.33 |  |  |
| Professionals/Managers/Technicians and Associate Professionals | 39.30 | 11.92 | 36.75 | 45.85 | 62.71 |  |  |
| **Weekly working hours (%)** |  |  |  |  |  | *** |  |
| Weekly hours missing (primarily due to not working) | 13.16 | 41.94 | 6.27 | 2.89 | 1.21 |  |  |
| Equal share of part- and full-time | 2.82 | 4.19 | 2.28 | 2.53 | 2.18 |  |  |
| Part-time (1-34 hrs/wk) | 32.35 | 37.75 | 34.47 | 34.66 | 21.55 |  |  |
| Full-time (> = 35 hrs/wk) | 51.67 | 16.11 | 56.98 | 59.93 | 75.06 |  |  |
| Average weekly working hours (mean) | 23.92 (n = 1747) | 6.11 (n = 453) | 30.73 (n = 338) | 27.37 (n = 547) | 33.43 (n = 409) | *** | 24 |
| **Health variables at age 35** |  |  |  |  |  |  |  |
| **Short Form Health Survey (SF-12)** |  |  |  |  |  |  |  |
| Physical function (0-100) | 49.28 (n = 1210) | 45.10 (n = 306) | 50.74 (n = 232) | 49.50 (n = 321) | 51.78 (n = 351) | *** | 561 |
| Mental function (0-100) | 48.93 (n = 1210) | 46.17 (n = 306) | 50.66 (n = 232) | 49.36 (n = 321) | 49.79 (n = 351) | *** | 561 |
| **Self-assessed general poor/fair health** | 14.10 | 25.71 | 8.94 | 12.35 | 8.78 | *** | 544 |
| **GHQ-Likert score (0-36; the higher, the worse)** | 12.11 (n = 1212) | 13.43 (n = 307) | 11.30 (n = 234) | 11.82 (n = 320) | 11.76 (n = 351) | *** | 559 |
| **Psychological distress (GHQ-Likert score >13)** | 28.47 | 36.81 | 23.93 | 26.56 | 25.93 | ** | 559 |

*Note*. NW: not working; ST: standard daytime hours; other NST: non-daytime hours other than evenings/nights (e.g., weekends, irregular). Bivariate statistical significant tests were done by Chi-square test for categorical variables or ANOVA for continuous variables. * *p* < .05, ** *p* < .01, *** *p* < .001.

|  | **By the schedule patterns between ages 35-44** | | | | | | **Sig.** | **# missing cases** |
| --- | --- | --- | --- | --- | --- | --- | --- | --- |
| **UKHLS (United Kingdom)** | **Total (n = 3363)** | **Mainly NW to some ST+NST (n = 720, 21.41%)** | **Mainly Evenings to volatile (n = 360, 10.7%)** | **Mainly other NST to ST+NST (n = 588, 17.48%)** | **Mainly ST to missing (n = 757, 22.51%)** | **Mainly ST (n = 938, 27.89%)** |  |  |
| **Gender (%)** |  |  |  |  |  |  | *** |  |
| Female | 60.18 | 86.53 | 52.22 | 47.62 | 55.61 | 54.58 |  |  |
| Male | 39.82 | 13.47 | 47.78 | 52.38 | 44.39 | 45.42 |  |  |
| **Ethnicity (%)** |  |  |  |  |  |  | *** | 1 |
| White | 77.87 | 63.89 | 77.72 | 80.78 | 79.52 | 85.50 |  |  |
| Mixed | 1.67 | 2.36 | 1.95 | 0.68 | 1.59 | 1.71 |  |  |
| Asian | 16.03 | 26.94 | 15.88 | 14.29 | 14.27 | 10.23 |  |  |
| Black | 3.39 | 5.56 | 3.34 | 3.23 | 3.04 | 2.13 |  |  |
| Other | 1.04 | 1.25 | 1.11 | 1.02 | 1.59 | 0.43 |  |  |
| **Background at age 25** |  |  |  |  |  |  |  |  |
| **Education (%)** |  |  |  |  |  |  | *** | 25 |
| Low education level | 4.34 | 12.73 | 4.49 | 1.37 | 1.99 | 1.71 |  |  |
| Medium education level | 44.04 | 51.77 | 44.66 | 53.33 | 39.12 | 36.11 |  |  |
| High education level | 51.62 | 35.50 | 50.84 | 45.30 | 58.89 | 62.18 |  |  |
| **Relationship status (%)** |  |  |  |  |  |  | *** |  |
| Not-partnered | 11.48 | 17.36 | 9.44 | 11.22 | 11.89 | 7.57 |  |  |
| Partnered | 88.52 | 82.64 | 90.56 | 88.78 | 88.11 | 92.43 |  |  |
| **Parenthood status (%)** |  |  |  |  |  |  | *** |  |
| No | 11.72 | 5.28 | 13.33 | 13.27 | 13.34 | 13.75 |  |  |
| Yes | 88.28 | 94.72 | 86.67 | 86.73 | 86.66 | 86.25 |  |  |
| **Work variables between ages 25-34** |  |  |  |  |  |  |  |  |
| **Occupation (%)** |  |  |  |  |  |  | *** |  |
| Occupation missing (primarily due to not working) | 9.19 | 41.53 | 0.28 | 0.51 | 0.26 | 0.43 |  | 309 |
| Other occupations | 16.83 | 15.14 | 22.78 | 24.15 | 15.32 | 12.47 |  |  |
| Clerks/Service- and Sales-related Workers | 26.97 | 27.50 | 29.44 | 34.01 | 23.78 | 23.77 |  |  |
| Professionals/Managers/Technicians and Associate Professionals | 47.01 | 15.83 | 47.50 | 41.33 | 60.63 | 63.33 |  |  |
| **Weekly working hours (%)** |  |  |  |  |  |  | *** |  |
| Weekly hours missing (primarily due to not working) | 14.81 | 45.14 | 12.22 | 8.50 | 5.28 | 4.16 |  |  |
| Equal share of part- and full-time | 1.69 | 2.64 | 1.94 | 1.19 | 1.59 | 1.28 |  |  |
| Part-time (1-34 hrs/wk) | 35.44 | 37.22 | 39.72 | 31.46 | 36.59 | 34.01 |  |  |
| Full-time (> = 35 hrs/wk) | 48.05 | 15.00 | 46.11 | 58.84 | 56.54 | 60.55 |  |  |
| Average weekly working hours (mean) | 25.42 (n = 3227) | 6.32 (n = 720) | 29.46 (n = 324) | 32.01 (n = 547) | 29.24 (n = 731) | 32.11 (n = 905) | *** | 136 |
| **Health variables at age 35** |  |  |  |  |  |  |  |  |
| **Short Form Health Survey (SF-12)** |  |  |  |  |  |  |  |  |
| Physical function (0-100) | 48.92 (n = 2083) | 44.89 (n = 421) | 49.24 (n = 219) | 49.33 (n = 367) | 49.22 (n = 317) | 50.75 (n = 759) | *** | 1280 |
| Mental function (0-100) | 49.10 (n = 2083) | 46.63 (n = 421) | 49.19 (n = 219) | 50.23 (n = 367) | 48.52 (n = 317) | 50.14 (n = 759) | *** | 1280 |
| **Self-assessed general poor/fair health** | 18.07 | 33.49 | 17.19 | 11.05 | 18.87 | 12.60 | *** | 1255 |
| **GHQ-Likert score (0-36; the higher, the worse)** | 12.00 (n = 2095) | 13.05 (n = 429) | 12.09 (n = 220) | 11.36 (n = 369) | 12.35 (n = 317) | 11.54 (n = 760) | *** | 1268 |
| **Psychological distress (GHQ-Likert score >13)** | 26.73 | 30.54 | 24.09 | 24.66 | 29.97 | 25.00 |  | 1268 |

*Note*. NW: not working; ST: standard daytime hours; other NST: non-daytime hours other than evenings/nights (e.g., weekends, irregular). Bivariate statistical significant tests were done by Chi-square test for categorical variables or ANOVA for continuous variables. * *p* < .05, ** *p* < .01, *** *p* < .001.

|  | **By the schedule patterns between ages 45-54** | | | | | **Sig.** | **# missing cases** |
| --- | --- | --- | --- | --- | --- | --- | --- |
| **UKHLS (United Kingdom)** | **Total (n = 2671)** | **Mainly NW (n = 309, 11.57%)** | **Mainly ST + some NST (n = 837, 31.34%)** | **Mainly other NST + Evenings (n = 470, 17.6%)** | **Mainly ST (n = 1055, 39.5%)** |  |  |
| **Gender (%)** |  |  |  |  |  | *** |  |
| Female | 54.47 | 73.79 | 53.17 | 43.62 | 54.69 |  |  |
| Male | 45.53 | 26.21 | 46.83 | 56.38 | 45.31 |  |  |
| **Ethnicity (%)** |  |  |  |  |  | *** | 2 |
| White | 82.54 | 64.40 | 82.68 | 78.72 | 89.46 |  |  |
| Mixed | 1.35 | 0.65 | 1.43 | 1.91 | 1.23 |  |  |
| Asian | 10.72 | 29.77 | 10.27 | 11.91 | 4.94 |  |  |
| Black | 4.46 | 3.56 | 5.02 | 6.17 | 3.51 |  |  |
| Other | 0.94 | 1.62 | 0.60 | 1.28 | 0.85 |  |  |
| **Background at age 25** |  |  |  |  |  |  |  |
| **Education (%)** |  |  |  |  |  | *** | 20 |
| Low education level | 4.87 | 19.61 | 3.25 | 5.35 | 1.62 |  |  |
| Medium education level | 45.98 | 54.90 | 46.39 | 51.18 | 40.74 |  |  |
| High education level | 49.15 | 25.49 | 50.36 | 43.47 | 57.63 |  |  |
| **Relationship status (%)** |  |  |  |  |  | * |  |
| Not-partnered | 12.77 | 17.48 | 11.83 | 14.47 | 11.37 |  |  |
| Partnered | 87.23 | 82.52 | 88.17 | 85.53 | 88.63 |  |  |
| **Parenthood status (%)** |  |  |  |  |  |  |  |
| No | 2.58 | 3.24 | 2.27 | 3.19 | 2.37 |  |  |
| Yes | 97.42 | 96.76 | 97.73 | 96.81 | 97.63 |  |  |
| **Work variables between ages 25-34** |  |  |  |  |  |  |  |
| **Occupation (%)** |  |  |  |  |  | *** |  |
| Occupation missing (primarily due to not working) | 8.99 | 74.76 | 0.36 | 0.85 | 0.19 |  | 240 |
| Other occupations | 18.46 | 7.77 | 23.06 | 30.00 | 12.80 |  |  |
| Clerks/Service- and Sales-related Workers | 26.51 | 11.33 | 30.47 | 28.09 | 27.11 |  |  |
| Professionals/Managers/Technicians and Associate Professionals | 46.05 | 6.15 | 46.12 | 41.06 | 59.91 |  |  |
| **Weekly working hours (%)** |  |  |  |  |  | *** |  |
| Weekly hours missing (primarily due to not working) | 17.90 | 78.32 | 10.63 | 18.72 | 5.59 |  |  |
| Equal share of part- and full-time | 1.05 | 0.32 | 1.55 | 1.49 | 0.66 |  |  |
| Part-time (1-34 hrs/wk) | 76.49 | 21.04 | 81.48 | 71.49 | 91.00 |  |  |
| Full-time (> = 35 hrs/wk) | 4.57 | 0.32 | 6.33 | 8.30 | 2.75 |  |  |
| Average weekly working hours (mean) | 26.48 (n = 2505) | 1.30 (n = 309) | 27.22 (n = 784) | 30.73 (n = 406) | 31.92 (n = 1006) | *** | 166 |
| **Health variables at age 35** |  |  |  |  |  |  |  |
| **Short Form Health Survey (SF-12)** |  |  |  |  |  |  |  |
| Physical function (0-100) | 48.41 (n = 1588) | 38.79 (n = 165) | 49.18 (n = 487) | 48.28 (n = 287) | 50.35 (n = 649) | *** | 1083 |
| Mental function (0-100) | 49.14 (n = 1588) | 41.7 (n = 165) | 49.72 (n = 487) | 50.44 (n = 287) | 50.02 (n = 649) | *** | 1083 |
| **Self-assessed general poor/fair health** | 20.56 | 55.29 | 17.14 | 18.24 | 15.16 | *** | 1056 |
| **GHQ-Likert score (0-36; the higher, the worse)** | 11.82 (n = 1599) | 15.21 (n = 164) | 11.56 (n = 491) | 10.93 (n = 292) | 11.55 (n = 652) | *** | 1072 |
| **Psychological distress (GHQ-Likert score >13)** | 25.45 | 45.73 | 25.87 | 20.21 | 22.39 | *** | 1072 |

*Note*. NW: not working; ST: standard daytime hours; other NST: non-daytime hours other than evenings/nights (e.g., weekends, irregular). Bivariate statistical significant tests were done by Chi-square test for categorical variables or ANOVA for continuous variables. * *p* < .05, ** *p* < .01, *** *p* < .001.

|  | **By the schedule patterns between ages 25-34** | | | | | **Sig.** | **# missing cases** |
| --- | --- | --- | --- | --- | --- | --- | --- |
| **NLSY79 (United States)** | **Total (n = 6497)** | **Volatile to mainly NW (n = 1066, 16.41%)** | **Volatile to mainly other NST (n = 849, 13.07%)** | **Volatile (n = 1634, 25.15%)** | **Mainly ST (n = 2948, 45.37%)** |  |  |
| **Gender (%)** |  |  |  |  |  | *** |  |
| Female | 56.09 | 82.74 | 50.65 | 55.26 | 48.47 |  |  |
| Male | 43.91 | 17.26 | 49.35 | 44.74 | 51.53 |  |  |
| **Race-Ethnicity (%)** |  |  |  |  |  | *** |  |
| Non-Hispanic White | 48.70 | 40.34 | 56.89 | 44.37 | 51.76 |  |  |
| Non-Hispanic Black | 29.11 | 35.55 | 24.85 | 33.84 | 25.37 |  |  |
| Hispanic | 21.07 | 23.26 | 17.20 | 20.69 | 21.61 |  |  |
| Others | 1.12 | 0.84 | 1.06 | 1.10 | 1.26 |  |  |
| **Background around age 25** |  |  |  |  |  |  |  |
| **Education (%)** |  |  |  |  |  | *** |  |
| Low education level | 21.01 | 35.55 | 16.84 | 22.28 | 16.25 |  |  |
| Medium education level | 64.97 | 56.00 | 69.26 | 68.91 | 64.79 |  |  |
| High education level | 14.02 | 8.44 | 13.90 | 8.81 | 18.96 |  |  |
| **Relationship status (%)** |  |  |  |  |  | ** | 14 |
| Not-partnered | 46.20 | 51.04 | 44.64 | 45.75 | 45.15 |  |  |
| Partnered | 53.80 | 48.96 | 55.36 | 54.25 | 54.85 |  |  |
| **Parenthood status (%)** |  |  |  |  |  | *** | 14 |
| No | 42.84 | 31.17 | 43.82 | 37.38 | 49.76 |  |  |
| Yes | 57.16 | 68.83 | 56.18 | 62.62 | 50.24 |  |  |
| **Work variables between ages 25-34** |  |  |  |  |  |  |  |
| **Occupation (%)** |  |  |  |  |  | *** |  |
| Occupation missing (primarily due to not working) | 3.83 | 20.83 | 0.24 | 1.04 | 0.27 |  | 249 |
| Other occupations | 33.40 | 22.05 | 29.80 | 34.64 | 37.86 |  |  |
| Clerks/Service- and Sales-related Workers | 43.39 | 49.44 | 46.17 | 48.78 | 37.42 |  |  |
| Professionals/Managers/Technicians and Associate Professionals | 19.38 | 7.69 | 23.79 | 15.54 | 24.46 |  |  |
| **Weekly working hours (%)** |  |  |  |  |  | *** |  |
| Weekly hours missing (primarily due to not working) | 4.37 | 24.20 | 0.35 | 0.86 | 0.31 |  |  |
| Equal share of part- and full-time | 3.49 | 7.41 | 3.53 | 4.47 | 1.53 |  |  |
| Part-time (1-34 hrs/wk) | 13.13 | 24.67 | 19.43 | 15.12 | 6.04 |  |  |
| Full-time (> = 35 hrs/wk) | 79.01 | 43.71 | 76.68 | 79.56 | 92.13 |  |  |
| Average weekly working hours (mean) | 34.70 (n = 6497) | 12.77 (n = 1066) | 38.82 (n = 849) | 35.89 (n = 1634) | 40.78 (n = 2948) | *** |  |
| **Health variables at age 40** |  |  |  |  |  |  |  |
| **Short Form Health Survey (SF-12)** |  |  |  |  |  |  |  |
| Physical function (0-100) | 51.89 (n = 5903) | 49.23 (n = 980) | 52.02 (n = 782) | 51.80 (n = 1408) | 52.86 (n = 2733) | *** | 594 |
| Mental function (0-100) | 52.88 (n = 5903) | 50.76 (n = 980) | 53.35 (n = 782) | 52.66 (n = 1408) | 53.62 (n = 2733) | *** | 594 |
| Self-assessed general poor/fair health | 13.67 | 23.89 | 13.74 | 14.42 | 9.58 | *** | 563 |
| **CES-D score** | 3.41  (n = 5870) | 4.70  (n = 977) | 3.32  (n = 782) | 3.67  (n = 1402) | 2.85  (n =2718) | *** |  |
| At risk of clinical depression (CES-D score >=8) | 14.65 | 23.34 | 13.94 | 16.12 | 10.96 | *** | 618 |

*Note*. NW: not working; ST: standard daytime hours; NST: non-day time hours other than evenings/nights (e.g., weekends, irregular). Bivariate statistical significant tests were done by Chi-square test for categorical variables or ANOVA for continuous variables. * *p* < .05, ** *p* < .01, *** *p* < .001.

|  | **By the schedule patterns between ages 35-44** | | | | | **Sig.** | **# missing cases** |
| --- | --- | --- | --- | --- | --- | --- | --- |
| **NLSY79 (United States)** | **Total (n = 6352)** | **Mainly NW to some ST (n = 829, 13.05%)** | **ST + NST (n = 2734, 43.04%)** | **Mainly other NST (n = 589, 9.27%)** | **ST only (n = 2200, 34.63%)** |  |  |
| **Gender (%)** |  |  |  |  |  | *** |  |
| Female | 57.65 | 83.11 | 55.96 | 52.29 | 51.59 |  |  |
| Male | 42.35 | 16.89 | 44.04 | 47.71 | 48.41 |  |  |
| **Race-Ethnicity (%)** |  |  |  |  |  | *** |  |
| Non-Hispanic White | 50.11 | 41.01 | 46.82 | 60.95 | 54.73 |  |  |
| Non-Hispanic Black | 28.04 | 34.98 | 31.49 | 22.92 | 22.50 |  |  |
| Hispanic | 20.69 | 23.16 | 20.59 | 15.28 | 21.32 |  |  |
| Others | 1.16 | 0.84 | 1.10 | 0.85 | 1.45 |  |  |
| **Background around age 35** |  |  |  |  |  |  |  |
| **Education (%)** |  |  |  |  |  | *** |  |
| Low education level | 17.10 | 30.04 | 17.37 | 11.38 | 13.41 |  |  |
| Medium education level | 62.45 | 57.06 | 65.91 | 65.20 | 59.45 |  |  |
| High education level | 20.45 | 12.91 | 16.72 | 23.43 | 27.14 |  |  |
| **Relationship status (%)** |  |  |  |  |  | *** |  |
| Not-partnered | 31.33 | 42.10 | 34.20 | 27.67 | 24.68 |  |  |
| Partnered | 68.67 | 57.90 | 65.80 | 72.33 | 75.32 |  |  |
| **Parenthood status (%)** |  |  |  |  |  | *** |  |
| No | 11.76 | 6.76 | 12.22 | 14.77 | 12.27 |  |  |
| Yes | 88.24 | 93.24 | 87.78 | 85.23 | 87.73 |  |  |
| **Work variables between ages 35-44** |  |  |  |  |  |  |  |
| **Occupation (%)** |  |  |  |  |  | *** |  |
| Occupation missing (primarily due to not working) | 4.52 | 31.12 | 1.06 | 0.00 | 0.00 |  | 287 |
| Other occupations | 27.47 | 14.72 | 30.76 | 27.33 | 28.23 |  |  |
| Clerks/Service- and Sales-related Workers | 38.71 | 41.62 | 42.79 | 40.24 | 32.14 |  |  |
| Professionals/Managers/Technicians and Associate Professionals | 29.30 | 12.55 | 25.38 | 32.43 | 39.64 |  |  |
| **Weekly working hours (%)** |  |  |  |  |  | *** |  |
| Weekly hours missing (primarily due to not working) | 5.64 | 34.86 | 2.30 | 0.51 | 0.14 |  |  |
| Equal share of part- and full-time | 2.79 | 5.67 | 2.96 | 3.06 | 1.41 |  |  |
| Part-time (1-34 hrs/wk) | 12.22 | 20.02 | 13.31 | 15.79 | 6.95 |  |  |
| Full-time (> = 35 hrs/wk) | 79.36 | 39.45 | 81.42 | 80.65 | 91.50 |  |  |
| Average weekly working hours (mean) | 36.64 (n = 6348) | 14.24 (n = 829) | 38.58 (n = 2731) | 43.80 (n = 588) | 40.77 (n = 2200) | *** | 4 |
| **Health variables at age 50** |  |  |  |  |  |  |  |
| **Short Form Health Survey (SF-12)** |  |  |  |  |  |  |  |
| Physical function (0-100) | 49.36 (n = 5572) | 45.30 (n = 705) | 49.04 (n = 2373) | 50.11 (n = 533) | 51.02 (n = 1961) | *** | 780 |
| Mental function (0-100) | 53.01 (n = 5572) | 50.36 (n = 705) | 52.93 (n = 2373) | 53.39 (n = 533) | 53.97 ( n = 1961) | *** | 780 |
| Self-assessed general poor/fair health | 19.29 | 32.54 | 19.97 | 17.29 | 14.25 | *** | 733 |
| **CES-D score** | 3.81  (n = 5575) | 5.16  (n = 705) | 3.96  (n = 2373) | 3.70  (n= 236) | 3.19  (n = 1961) | *** | 777 |
| At risk of clinical depression (CES-D score >=8) | 16.75 | 25.67 | 18.04 | 15.30 | 12.39 | *** | 777 |

*Note*. NW: not working; ST: standard daytime hours; other NST: non-daytime hours other than evenings/nights (e.g., weekends, irregular). Bivariate statistical significant tests were done by Chi-square test for categorical variables or ANOVA for continuous variables. * *p* < .05, ** *p* < .01, *** *p* < .001.

|  | **By the schedule patterns between ages 45-54** | | | | | | | | **Sig.** | **# missing cases** |
| --- | --- | --- | --- | --- | --- | --- | --- | --- | --- | --- |
| **NLSY79 (United States)** | **Total (n = 4919)** | **Mainly NW (n = 401, 8.15%)** | **ST+NST to mainly ST (n = 889, 18.07%)** | **ST to other NST (n = 337, 6.85%)** | **Other NST to ST (n = 311, 6.32%)** | **Mainly Evenings/Nights (n = 556, 11.30%)** | **Mainly other NST (n = 504, 10.25%)** | **ST only (n = 1921, 39.05%)** |  |  |
| **Gender (%)** |  |  |  |  |  |  |  |  | *** |  |
| Female | 57.71 | 84.04 | 62.54 | 59.05 | 59.81 | 49.28 | 54.96 | 52.58 |  |  |
| Male | 42.29 | 15.96 | 37.46 | 40.95 | 40.19 | 50.72 | 45.04 | 47.42 |  |  |
| **Race-Ethnicity (%)** |  |  |  |  |  |  |  |  | *** |  |
| Non-Hispanic White | 50.80 | 46.38 | 43.76 | 50.15 | 59.49 | 39.39 | 54.96 | 55.91 |  |  |
| Non-Hispanic Black | 27.34 | 31.42 | 31.50 | 26.41 | 22.83 | 42.27 | 25.00 | 21.76 |  |  |
| Hispanic | 20.72 | 21.45 | 23.51 | 22.85 | 16.40 | 17.63 | 18.65 | 21.03 |  |  |
| Others | 1.14 | 0.75 | 1.24 | 0.59 | 1.29 | 0.72 | 1.39 | 1.30 |  |  |
| **Background around age 45** |  |  |  |  |  |  |  |  |  |  |
| **Education (%)** |  |  |  |  |  |  |  |  | *** |  |
| Low education level | 12.46 | 24.69 | 15.75 | 12.76 | 10.61 | 13.85 | 7.54 | 9.53 |  |  |
| Medium education level | 61.70 | 55.36 | 61.98 | 62.91 | 61.41 | 73.56 | 65.87 | 58.20 |  |  |
| High education level | 25.84 | 19.95 | 22.27 | 24.33 | 27.97 | 12.59 | 26.59 | 32.27 |  |  |
| **Relationship status (%)** |  |  |  |  |  |  |  |  | *** | 10 |
| Not-partnered | 30.35 | 38.60 | 35.33 | 31.16 | 29.58 | 43.17 | 27.29 | 23.43 |  |  |
| Partnered | 69.65 | 61.40 | 64.67 | 68.84 | 70.42 | 56.83 | 72.71 | 76.57 |  |  |
| **Parenthood status (%)** |  |  |  |  |  |  |  |  | *** | 10 |
| No | 8.39 | 8.27 | 9.51 | 10.68 | 9.65 | 12.05 | 10.36 | 5.73 |  |  |
| Yes | 91.61 | 91.73 | 90.49 | 89.32 | 90.35 | 87.95 | 89.64 | 94.27 |  |  |
| **Work variables between ages 45-54** |  |  |  |  |  |  |  |  |  |  |
| **Occupation (%)** |  |  |  |  |  |  |  |  | *** |  |
| Occupation missing (primarily due to not working) | 8.54 | 49.88 | 11.59 | 4.15 | 2.89 | 4.50 | 7.14 | 1.72 |  | 420 |
| Other occupations | 21.33 | 7.23 | 22.05 | 20.77 | 18.33 | 32.01 | 21.83 | 21.29 |  |  |
| Clerks/Service- and Sales-related Workers | 38.56 | 31.92 | 40.16 | 41.84 | 39.55 | 43.35 | 43.45 | 35.81 |  |  |
| Professionals/Managers/Technicians and Associate Professionals | 31.57 | 10.97 | 26.21 | 33.23 | 39.23 | 20.14 | 27.58 | 41.18 |  |  |
| **Weekly working hours (%)** |  |  |  |  |  |  |  |  | *** |  |
| Weekly hours missing (primarily due to not working) | 8.97 | 53.87 | 10.57 | 5.04 | 3.54 | 5.76 | 7.34 | 1.77 |  |  |
| Equal share of part- and full-time | 2.18 | 3.24 | 3.60 | 1.78 | 2.89 | 1.80 | 2.58 | 1.25 |  |  |
| Part-time (1-34 hrs/wk) | 11.32 | 20.45 | 13.84 | 15.13 | 17.04 | 7.01 | 16.07 | 6.66 |  |  |
| Full-time (> = 35 hrs/wk) | 77.54 | 22.44 | 71.99 | 78.04 | 76.53 | 85.43 | 74.01 | 90.32 |  |  |
| Average weekly working hours (mean) | 35.44 (n = 4918) | 10.19 (n = 401) | 33.07 (n = 889) | 37.36 (n = 337) | 37.25 (n = 311) | 37.56 (n = 556) | 38.15 (n = 503) | 39.87 (n = 1921) | *** | 1 |
| **Health variables at age 60** |  |  |  |  |  |  |  |  |  |  |
| **Short Form Health Survey (SF-12)** |  |  |  |  |  |  |  |  |  |  |
| Physical function (0-100) | 46.71 (n = 3040) | 39.72 (n = 228) | 45.84 (n = 490) | 46.13 (n = 216) | 47.52 (n = 207) | 45.92 (n = 331) | 45.29 (n = 322) | 48.87 (n = 1246) | *** | 1879 |
| Mental function (0-100) | 53.22 (n = 3040) | 50.87 (n = 228) | 52.93 (n = 490) | 52.69 (n = 216) | 53.54 (n = 207) | 52.35 (n = 331) | 52.99 (n = 322) | 54.09 (n = 1246) | *** | 1879 |
| Self-assessed general poor/fair health | 22.71 | 37.50 | 25.86 | 24.20 | 19.23 | 27.81 | 24.15 | 17.33 | *** | 1846 |
| **CES-D score** | 3.53  (n = 2536) | 4.92  (n = 181) | 3.74  (n = 401) | 4.07  (n = 191) | 3.54  (n = 184) | 3.84  (n = 277) | 4.03  (n = 260) | 2.89  (n = 1042) | *** | 2383 |
| At risk of clinical depression (CES-D score >=8) | 15.89 | 27.62 | 17.96 | 19.37 | 16.30 | 16.97 | 21.92 | 10.56 | *** | 2383 |

*Note*. NW: not working; ST: standard daytime hours; other NST: non-daytime hours other than evenings/nights (e.g., weekends, irregular). Bivariate statistical significant tests were done by Chi-square test for categorical variables or ANOVA for continuous variables. * *p* < .05, ** *p* < .01, *** *p* < .001.
