# Supplementary material for: Longitudinal employment patterns and parental health: A cross-country look
Source: PLoS One. 2026 Jun 5;21(6):e0350945. doi: 10.1371/journal.pone.0350945 (PMC13240889; doi:10.1371/journal.pone.0350945)
Supplement: S4 Table — (DOCX) [file pone.0350945.s004.docx]

**S4. Table. Parental Health Outcomes by Age and Country**

| **Australia: HILDA Health Outcomes at Age 35** | **SF36-Physical Health** | **SF36-Mental Health** | **Self-assessed Poor/Fair Health** | **Kessler-10 Psychological Distress Score** | **At risk of psychological distress (Kessler-10>=25)** |
| --- | --- | --- | --- | --- | --- |
| Work schedule patterns between ages 25-34 (ref: mainly ST) |  |  |  |  |  |
| Mainly NW | -3.488^**^ | -3.570^**^ | 0.945^**^ | 4.000^***^ | 1.324^***^ |
|  | (-2.73) | (-2.73) | (2.68) | (4.82) | (4.30) |
|  |  |  |  |  |  |
| NW + ST | -1.522^*^ | -2.824^***^ | 0.654^**^ | 1.966^***^ | 0.740^***^ |
|  | (-2.28) | (-4.12) | (2.95) | (4.53) | (3.74) |
|  |  |  |  |  |  |
| Other NST + ST | 0.248 | -0.364 | -0.0116 | -0.156 | -0.159 |
|  | (0.41) | (-0.58) | (-0.05) | (-0.39) | (-0.73) |
| Sociodemographic characteristics |  |  |  |  |  |
| Male (vs. female) | -0.650 | 0.198 | 0.266 | 0.264 | 0.182 |
|  | (-1.13) | (0.34) | (1.26) | (0.71) | (0.99) |
| Race-immigrant (ref: non-indigenous Australian) |  |  |  |  |  |
| Indigenous/Torres Strait Islander Australian | -2.733^*^ | -1.433 | 0.178 | 1.161 | 0.0800 |
|  | (-2.20) | (-1.13) | (0.53) | (1.43) | (0.26) |
|  |  |  |  |  |  |
| Other English-speaking country | -0.621 | -1.096 | 0.0431 | -0.152 | 0.174 |
|  | (-0.62) | (-1.07) | (0.12) | (-0.24) | (0.60) |
|  |  |  |  |  |  |
| Non-English-speaking country | -0.245 | 0.490 | -0.357 | 0.300 | 0.271 |
|  | (-0.30) | (0.58) | (-1.11) | (0.57) | (1.09) |
| Education around age 25 (ref: high) |  |  |  |  |  |
| Low Education Level | -1.288 | -0.507 | 0.183 | 1.276^*^ | 0.774^**^ |
|  | (-1.67) | (-0.64) | (0.70) | (2.55) | (3.23) |
|  |  |  |  |  |  |
| Medium Education Level | -0.715 | -0.510 | 0.126 | 1.252^***^ | 0.823^***^ |
|  | (-1.29) | (-0.89) | (0.60) | (3.47) | (4.19) |
|  |  |  |  |  |  |
| Not-partnered around age 25 (vs. partnered) | -0.891 | -0.208 | 0.301 | 0.113 | -0.0983 |
|  | (-1.67) | (-0.38) | (1.74) | (0.33) | (-0.61) |
| Primary occupation between ages 25-34 (ref: professional/managerial) |  |  |  |  |  |
| Occupation missing (primarily due to not working) | -1.705 | -2.344 | 0.787 | 0.606 | 0.395 |
|  | (-1.10) | (-1.48) | (1.84) | (0.60) | (1.07) |
|  |  |  |  |  |  |
| Other Occupations | -1.753^*^ | -0.600 | 0.354 | 1.080^*^ | 0.412^*^ |
|  | (-2.58) | (-0.86) | (1.51) | (2.45) | (2.10) |
|  |  |  |  |  |  |
| Clerks/Service-related workers/Sales-related workers | -0.948 | -0.0332 | 0.231 | 0.269 | 0.149 |
|  | (-1.54) | (-0.05) | (1.03) | (0.67) | (0.73) |
| Primary weekly working hours between ages 25-34 (ref: full-time) |  |  |  |  |  |
| Equal share of part- and full-time | 0.196 | -3.810^**^ | 0.0195 | 2.056^*^ | 0.724^*^ |
|  | (0.15) | (-2.88) | (0.04) | (2.43) | (2.17) |
|  |  |  |  |  |  |
| Part-time (1-34 hrs/wk) | -0.0565 | 0.280 | 0.517^*^ | -0.241 | -0.244 |
|  | (-0.09) | (0.43) | (2.37) | (-0.58) | (-1.19) |
|  |  |  |  |  |  |
| Constant | 51.55^***^ | 50.99^***^ | -3.131^***^ | 14.51^***^ | -3.010^***^ |
|  | (91.40) | (88.11) | (-13.62) | (39.24) | (-13.96) |
| Observations | 1880 | 1886 | 1880 | 1923 | 1923 |

*Note.* NW: not working; ST: standard daytime hours; other NST: non-daytime hours other than evenings/nights (e.g., weekends, irregular). *t-*statistics in parentheses.

^*^ *p* < 0.05, ^**^ *p* < 0.01, ^***^ *p* < 0.001.

| **Australia: HILDA Health Outcomes at Age 45** | **SF36-Physical Health** | **SF36-Mental Health** | **Self-assessed Poor/Fair Health** | **Kessler-10 Psychological Distress Score** | **At risk of psychological distress (Kessler-10>=25)** |
| --- | --- | --- | --- | --- | --- |
| Work schedule patterns between ages 35-44 (ref: mainly ST) |  |  |  |  |  |
| Mainly NW | -4.500^***^ | -2.539^*^ | 0.772^*^ | 2.521^**^ | 0.918^**^ |
|  | (-3.55) | (-2.03) | (2.40) | (3.12) | (2.63) |
|  |  |  |  |  |  |
| Volatile to mainly ST | -0.798 | -0.527 | 0.0832 | 0.339 | 0.354 |
|  | (-0.93) | (-0.62) | (0.32) | (0.62) | (1.29) |
|  |  |  |  |  |  |
| Mainly other NST | -1.433 | -0.159 | 0.130 | 0.315 | 0.215 |
|  | (-1.19) | (-0.13) | (0.36) | (0.41) | (0.55) |
|  |  |  |  |  |  |
| ST only | 0.729 | 1.091 | -0.345 | -0.931 | -0.613 |
|  | (0.94) | (1.42) | (-1.33) | (-1.88) | (-1.94) |
| Sociodemographic characteristics |  |  |  |  |  |
| Male (vs. female) | -1.182 | 0.458 | -0.0143 | -0.307 | 0.206 |
|  | (-1.54) | (0.60) | (-0.06) | (-0.63) | (0.78) |
| Race-immigrant (ref: non-indigenous Australian) |  |  |  |  |  |
| Indigenous/Torres Strait Islander Australian | -6.243^**^ | -4.227^*^ | 0.711 | 1.583 | 0.394 |
|  | (-2.88) | (-2.02) | (1.50) | (1.22) | (0.79) |
|  |  |  |  |  |  |
| Other English-speaking country | -0.571 | -0.274 | -0.0335 | 0.879 | 0.0831 |
|  | (-0.51) | (-0.25) | (-0.09) | (1.23) | (0.22) |
|  |  |  |  |  |  |
| Non-English-speaking country | -0.813 | -1.364 | 0.145 | 1.181 | 0.332 |
|  | (-0.84) | (-1.42) | (0.53) | (1.93) | (1.17) |
| Education around age 25 (ref: high) |  |  |  |  |  |
| Low Education Level | -1.141 | 0.117 | 0.433 | 0.690 | 0.323 |
|  | (-1.28) | (0.13) | (1.69) | (1.21) | (1.12) |
|  |  |  |  |  |  |
| Medium Education Level | -1.476^*^ | 0.303 | 0.256 | 0.787 | 0.423 |
|  | (-2.08) | (0.43) | (1.15) | (1.75) | (1.75) |
|  |  |  |  |  |  |
| Not-partnered around age 35 (vs. partnered) | -2.701^**^ | -2.914^**^ | 0.607^**^ | 1.883^**^ | 0.400 |
|  | (-2.90) | (-3.17) | (2.60) | (3.21) | (1.54) |
| Primary occupation between ages 35-44 (ref: professional/managerial) |  |  |  |  |  |
| Occupation missing (primarily due to not working) | -3.260 | -3.588^*^ | 0.644 | 2.185 | 0.706 |
|  | (-1.78) | (-1.99) | (1.46) | (1.88) | (1.49) |
|  |  |  |  |  |  |
| Other Occupations | 0.110 | 1.423 | 0.125 | -0.358 | 0.100 |
|  | (0.13) | (1.65) | (0.49) | (-0.65) | (0.37) |
|  |  |  |  |  |  |
| Clerks/Service-related workers/Sales-related workers | 1.780^*^ | 1.512 | -0.107 | -0.532 | -0.250 |
|  | (2.24) | (1.93) | (-0.44) | (-1.05) | (-0.91) |
| Primary weekly working hours between ages 35-44 (ref: full-time) |  |  |  |  |  |
| Equal share of part- and full-time | -1.805 | -5.298^**^ | 0.401 | 2.372^*^ | 0.971 |
|  | (-0.95) | (-2.82) | (0.79) | (1.97) | (1.94) |
|  |  |  |  |  |  |
| Part-time (1-34 hrs/wk) | -0.636 | -0.897 | 0.164 | 0.328 | 0.274 |
|  | (-0.78) | (-1.11) | (0.66) | (0.63) | (0.97) |
|  |  |  |  |  |  |
| Constant | 51.54^***^ | 50.13^***^ | -2.372^***^ | 15.16^***^ | -2.820^***^ |
|  | (57.89) | (56.90) | (-8.35) | (26.64) | (-8.64) |
| Observations | 1220 | 1223 | 1221 | 1208 | 1208 |

*Note.* NW: not working; ST: standard daytime hours; other NST: non-daytime hours other than evenings/nights (e.g., weekends, irregular). *t*-statistics in parentheses.

^*^ *p* < 0.05, ^**^ *p* < 0.01, ^***^ *p* < 0.001.

| **Australia: HILDA Health Outcomes at Age 55** | **SF36-Physical Health** | **SF36-Mental Health** | **Self-assessed Poor/Fair Health** | **Kessler-10 Psychological Distress score** | **At risk of psychological distress (Kessler-10>=25)** |
| --- | --- | --- | --- | --- | --- |
| Work schedule patterns between ages 45-54 (ref: mainly ST) |  |  |  |  |  |
| Mainly NW | -3.884^**^ | -4.198^**^ | 0.554 | 1.893^*^ | 0.526 |
|  | (-3.02) | (-3.21) | (1.75) | (2.37) | (1.38) |
|  |  |  |  |  |  |
| Volatile | -5.250^***^ | -2.768^**^ | 1.111^***^ | 2.076^***^ | 0.506 |
|  | (-5.34) | (-2.78) | (4.89) | (3.40) | (1.64) |
|  |  |  |  |  |  |
| ST + other NST | 0.686 | -0.000191 | -0.0268 | 0.356 | 0.0676 |
|  | (0.80) | (-0.00) | (-0.11) | (0.66) | (0.21) |
|  |  |  |  |  |  |
| Mainly other NST | -0.847 | 0.503 | 0.161 | 0.110 | -0.0168 |
|  | (-0.93) | (0.54) | (0.67) | (0.19) | (-0.05) |
|  |  |  |  |  |  |
| Mainly ST to missing | -1.204 | -0.195 | 0.474 | 1.071 | 0.611 |
|  | (-0.55) | (-0.09) | (0.89) | (0.78) | (0.94) |
|  |  |  |  |  |  |
| ST only | 1.798^**^ | 1.383^*^ | -0.411^*^ | -0.683 | -0.361 |
|  | (2.91) | (2.20) | (-2.21) | (-1.79) | (-1.45) |
| Sociodemographic characteristics |  |  |  |  |  |
| Male (vs. female) | -2.908^***^ | 0.223 | 0.482^**^ | -0.731^*^ | -0.257 |
|  | (-4.87) | (0.37) | (2.89) | (-1.98) | (-1.23) |
| Race-immigrant (ref: non-indigenous Australian) |  |  |  |  |  |
| Indigenous/Torres Strait Islander Australian | -0.0608 | 1.650 | -0.138 | 0.0598 | 0.00349 |
|  | (-0.03) | (0.93) | (-0.32) | (0.06) | (0.01) |
|  |  |  |  |  |  |
| Other English-speaking country | 1.367 | 0.365 | -0.224 | -0.268 | 0.0623 |
|  | (1.76) | (0.46) | (-1.00) | (-0.56) | (0.23) |
|  |  |  |  |  |  |
| Non-English-speaking country | 0.254 | 0.445 | -0.117 | 0.0444 | 0.0637 |
|  | (0.35) | (0.61) | (-0.58) | (0.10) | (0.26) |
| Education around age 25 (ref: high) |  |  |  |  |  |
| Low Education Level | -1.826^**^ | 0.0762 | 0.654^***^ | 0.362 | 0.250 |
|  | (-2.59) | (0.11) | (3.50) | (0.83) | (1.03) |
|  |  |  |  |  |  |
| Medium Education Level | -0.693 | 0.633 | 0.257 | 0.0409 | 0.120 |
|  | (-1.16) | (1.05) | (1.51) | (0.11) | (0.54) |
|  |  |  |  |  |  |
| Not-partnered around age 45 (vs. partnered) | -3.042^***^ | -2.194^**^ | 0.396^*^ | 2.088^***^ | 0.815^***^ |
|  | (-4.30) | (-3.05) | (2.18) | (4.75) | (3.99) |
| Primary occupation between ages 45-54 (ref: professional/managerial) |  |  |  |  |  |
| Occupation missing (primarily due to not working) | -3.350^*^ | -1.325 | 0.676 | 3.025^**^ | 1.038^*^ |
|  | (-2.00) | (-0.78) | (1.72) | (2.93) | (2.26) |
|  |  |  |  |  |  |
| Other Occupations | -0.805 | -0.843 | 0.0776 | 0.480 | 0.298 |
|  | (-1.07) | (-1.11) | (0.40) | (1.04) | (1.15) |
|  |  |  |  |  |  |
| Clerks/Service-related workers/Sales-related workers | 0.199 | -0.954 | -0.0602 | 0.246 | 0.164 |
|  | (0.30) | (-1.43) | (-0.32) | (0.61) | (0.68) |
| Primary weekly working hours between ages 45-54 (ref: full-time) |  |  |  |  |  |
| Equal share of part- and full-time | -0.829 | 0.557 | 0.581 | 0.507 | 0.124 |
|  | (-0.53) | (0.35) | (1.52) | (0.52) | (0.26) |
|  |  |  |  |  |  |
| Part-time (1-34 hrs/wk) | -1.044 | 0.766 | 0.0994 | -0.625 | -0.172 |
|  | (-1.53) | (1.11) | (0.52) | (-1.48) | (-0.72) |
|  |  |  |  |  |  |
| Constant | 52.21^***^ | 50.02^***^ | -2.226^***^ | 15.23^***^ | -2.607^***^ |
|  | (73.27) | (69.08) | (-10.61) | (34.37) | (-9.93) |
| Observations | 1699 | 1702 | 1698 | 1715 | 1715 |

*Note.* NW: not working; ST: standard daytime hours; other NST: non-daytime hours other than evenings/nights (e.g., weekends, irregular). *t*-statistics in parentheses.

^*^ *p* < 0.05, ^**^ *p* < 0.01, ^***^ *p* < 0.001.

| **Germany: SOEP Health Outcomes at Age 35** | **Sf12-Physical Function** | **SF12-Mental Function** | **Self-assessed Poor/Fair Health** |
| --- | --- | --- | --- |
| Work schedule patterns between ages 25-34 (ref: mainly ST) |  |  |  |
| Mainly NW to some ST+NST | -1.597 | -2.217 | 0.802^*^ |
|  | (-1.37) | (-1.91) | (2.26) |
|  |  |  |  |
| Volatile | 0.0624 | 0.522 | -0.0431 |
|  | (0.06) | (0.48) | (-0.11) |
|  |  |  |  |
| Volatile to mainly Evenings | -1.973 | -0.243 | 0.132 |
|  | (-1.87) | (-0.23) | (0.34) |
| Sociodemographic characteristics |  |  |  |
| Male (vs. female) | 0.763 | 3.318^**^ | 0.0269 |
|  | (0.75) | (3.25) | (0.08) |
| Immigrant generation (ref: not an immigrant) |  |  |  |
| First generation | -1.789 | -0.400 | 0.206 |
|  | (-1.56) | (-0.35) | (0.59) |
|  |  |  |  |
| Second generation | 0.612 | -0.736 | 0.330 |
|  | (0.41) | (-0.49) | (0.74) |
| Education around age 25 (ref: high) |  |  |  |
| Low Education Level | -2.468 | -1.584 | 0.941 |
|  | (-1.64) | (-1.05) | (1.81) |
|  |  |  |  |
| Medium Education Level | -0.660 | 0.00646 | 0.423 |
|  | (-0.58) | (0.01) | (0.91) |
|  |  |  |  |
| Not-partnered around age 25 (vs. partnered) | -0.352 | -0.505 | -0.0341 |
|  | (-0.43) | (-0.61) | (-0.12) |
| Primary occupation between ages 25-34 (ref: professional/managerial) |  |  |  |
| Occupation missing (primarily due to not working) | -1.757 | -1.189 | -0.181 |
|  | (-0.64) | (-0.43) | (-0.22) |
|  |  |  |  |
| Other occupations | -1.935 | -0.244 | 0.291 |
|  | (-1.70) | (-0.21) | (0.79) |
|  |  |  |  |
| Clerks/Service-related workers/Sales-related workers | -1.450 | -0.820 | 0.176 |
|  | (-1.48) | (-0.84) | (0.52) |
| Primary weekly working hours between ages 25-34 (ref: full-time) |  |  |  |
| Weekly hours missing (primarily due to not working) | -1.313 | 1.642 | 0.616 |
|  | (-0.42) | (0.53) | (0.72) |
|  |  |  |  |
| Equal share of part- and full-time | -0.943 | 2.255 | -0.824 |
|  | (-0.54) | (1.28) | (-1.07) |
|  |  |  |  |
| Part-time (1-34 hrs/wk) | -0.433 | 0.492 | 0.0956 |
|  | (-0.44) | (0.51) | (0.29) |
|  |  |  |  |
| Constant | 52.13^***^ | 49.63^***^ | -3.132^***^ |
|  | (46.29) | (44.02) | (-6.48) |
| Observations | 663 | 663 | 738 |

*Note.* NW: not working; ST: standard daytime hours; other NST: non-daytime hours other than evenings/nights (e.g., weekends, irregular). *t*-statistics in parentheses.

^*^ *p* < 0.05, ^**^ *p* < 0.01, ^***^ *p* < 0.001.

| **Germany: SOEP Health Outcomes at Age 45** | **Sf12-Physical Function** | **SF12-Mental Function** | **Self-assessed Poor/Fair Health** |
| --- | --- | --- | --- |
| Work schedule patterns between ages 35-44 (ref: mainly ST) |  |  |  |
| Mainly NW | -2.202 | -1.396 | 0.850^**^ |
|  | (-1.75) | (-1.13) | (2.76) |
|  |  |  |  |
| Volatile | -0.851 | -0.676 | 0.260 |
|  | (-1.29) | (-1.05) | (1.28) |
|  |  |  |  |
| Mainly Nights | -0.357 | -0.950 | 0.135 |
|  | (-0.41) | (-1.12) | (0.50) |
|  |  |  |  |
| Volatile to mainly ST | -0.487 | -0.726 | 0.365 |
|  | (-0.77) | (-1.16) | (1.91) |
| Sociodemographic characteristics |  |  |  |
| Male (vs. female) | -0.210 | 1.513^*^ | 0.238 |
|  | (-0.32) | (2.36) | (1.24) |
| Immigrant generation (ref: not an immigrant) |  |  |  |
| First generation | -3.940^***^ | 0.248 | 0.260 |
|  | (-3.51) | (0.23) | (0.91) |
|  |  |  |  |
| Second generation | -2.339^***^ | 0.316 | 0.337 |
|  | (-4.14) | (0.57) | (1.94) |
| Education around age 35 (ref: high) |  |  |  |
| Low Education Level | -1.105 | -0.391 | 0.325 |
|  | (-1.53) | (-0.55) | (1.73) |
|  |  |  |  |
| Medium Education Level | -0.552 | -0.790 | 0.447 |
|  | (-0.49) | (-0.71) | (1.67) |
|  |  |  |  |
| Not-partnered around age 35 (vs. partnered) | -2.616^***^ | -3.073^***^ | 0.829^***^ |
|  | (-3.46) | (-4.14) | (4.58) |
| Primary occupation between ages 35-44 (ref: professional/managerial) |  |  |  |
| Occupation missing (primarily due to not working) | 0.521 | -0.486 | 0.0803 |
|  | (0.23) | (-0.22) | (0.16) |
|  |  |  |  |
| Other occupations | -2.994^***^ | 0.0977 | 0.308 |
|  | (-4.25) | (0.14) | (1.58) |
|  |  |  |  |
| Clerks/Service-related workers/Sales-related workers | -2.376^***^ | 0.629 | 0.220 |
|  | (-3.65) | (0.98) | (1.19) |
| Primary weekly working hours between ages 35-44 (ref: full-time) |  |  |  |
| Weekly hours missing (primarily due to not working) | -2.944 | -2.822 | 0.720 |
|  | (-1.20) | (-1.18) | (1.34) |
|  |  |  |  |
| Equal share of part- and full-time | 2.403 | -5.394^***^ | 0.488 |
|  | (1.44) | (-3.30) | (1.18) |
|  |  |  |  |
| Part-time (1-34 hrs/wk) | -1.106 | -0.356 | 0.428^*^ |
|  | (-1.69) | (-0.55) | (2.29) |
|  |  |  |  |
| Constant | 53.29^***^ | 50.45^***^ | -3.075^***^ |
|  | (71.80) | (69.31) | (-12.87) |
| Observations | 1891 | 1891 | 2129 |

*Note.* NW: not working; ST: standard daytime hours; other NST: non-daytime hours other than evenings/nights (e.g., weekends, irregular). *t*-statistics in parentheses.

^*^ *p* < 0.05, ^**^ *p* < 0.01, ^***^ *p* < 0.001.

| **Germany: SOEP Health Outcomes at age 55** | **SF12-Physical Function** | **SF12-Mental Function** | **Self-assessed Poor/Fair Health** |
| --- | --- | --- | --- |
| Work schedule patterns between ages 45-54 (ref: mainly ST) |  |  |  |
| Mainly NW to some ST | -5.030^***^ | -1.377 | 0.704^***^ |
|  | (-5.64) | (-1.46) | (3.40) |
|  |  |  |  |
| Mainly Nights | -3.020^***^ | -0.516 | 0.448^*^ |
|  | (-3.45) | (-0.56) | (2.07) |
|  |  |  |  |
| Mainly Evenings | -1.810^*^ | -0.366 | -0.0359 |
|  | (-2.00) | (-0.38) | (-0.14) |
|  |  |  |  |
| Mainly other NST | -0.911 | 1.095 | -0.0220 |
|  | (-1.10) | (1.25) | (-0.09) |
|  |  |  |  |
| Mainly ST to ST+NST | -0.422 | 0.448 | 0.115 |
|  | (-0.53) | (0.53) | (0.53) |
| Sociodemographic characteristics |  |  |  |
| Male (vs. female) | -0.386 | 0.247 | 0.0942 |
|  | (-0.69) | (0.41) | (0.64) |
| Immigrant generation (ref: not an immigrant) |  |  |  |
| First generation | 1.366 | -0.161 | -0.0824 |
|  | (1.46) | (-0.16) | (-0.36) |
|  |  |  |  |
| Second generation | -1.477 | -0.473 | 0.635 |
|  | (-0.83) | (-0.25) | (1.71) |
| Education around age 45 (ref: high) |  |  |  |
| Low Education Level | -5.284^***^ | 0.909 | 0.523 |
|  | (-3.95) | (0.64) | (1.87) |
|  |  |  |  |
| Medium Education Level | -1.878^***^ | -0.0587 | 0.166 |
|  | (-3.35) | (-0.10) | (1.11) |
|  |  |  |  |
| Not-partnered around age 45 (vs. partnered) | -1.977^**^ | -2.174^**^ | 0.626^***^ |
|  | (-2.89) | (-3.01) | (3.98) |
| Primary occupation between ages 45-54 (ref: professional/managerial) |  |  |  |
| Occupation missing (primarily due to not working) | -3.603 | 0.150 | -0.0753 |
|  | (-1.31) | (0.05) | (-0.13) |
|  |  |  |  |
| Other occupations | -3.448^***^ | 0.401 | 0.555^**^ |
|  | (-4.84) | (0.53) | (3.12) |
|  |  |  |  |
| Clerks/Service-related workers/Sales-related workers | -1.754^*^ | -0.548 | 0.456^*^ |
|  | (-2.47) | (-0.73) | (2.53) |
| Primary weekly working hours between ages 45-54 (ref: full-time) |  |  |  |
| Weekly hours missing (primarily due to not working) | -1.192 | -0.312 | 1.109 |
|  | (-0.42) | (-0.10) | (1.87) |
|  |  |  |  |
| Equal share of part- and full-time | 0.620 | 0.530 | 0.538 |
|  | (0.26) | (0.21) | (0.93) |
|  |  |  |  |
| Part-time (1-34 hrs/wk) | 2.084 | 1.518 | 0.101 |
|  | (1.90) | (1.31) | (0.36) |
|  |  |  |  |
| Constant | 52.62^***^ | 50.50^***^ | -2.385^***^ |
|  | (84.34) | (76.59) | (-13.41) |
| Observations | 1507 | 1507 | 1791 |

*Note.* NW: not working; ST: standard daytime hours; other NST: non-daytime hours other than evenings/nights (e.g., weekends, irregular). *t*-statistics in parentheses.

^*^ *p* < 0.05, ^**^ *p* < 0.01, ^***^ *p* < 0.001.

| **UK: UKHLS Health Outcomes at Age 35** | **SF12-Physical Function** | **SF12-Mental Function** | **Self-assessed Poor/Fair Health** | **GHQ12-Poor Subjective Well-Being** | **Psychological distress (GHQ-12>13)** |
| --- | --- | --- | --- | --- | --- |
| Work schedule patterns between ages 25-34 (ref: mainly ST) |  |  |  |  |  |
| Mainly NW to some ST+NST | -2.489^*^ | -1.797 | 0.524 | 0.660 | 0.127 |
|  | (-2.47) | (-1.81) | (1.77) | (1.17) | (0.57) |
|  |  |  |  |  |  |
| Volatile to mainly other NST to volatile | -0.388 | 1.039 | -0.202 | -0.504 | -0.124 |
|  | (-0.44) | (1.19) | (-0.65) | (-1.02) | (-0.61) |
|  |  |  |  |  |  |
| Mainly ST + some NST | -1.660^*^ | -0.0926 | 0.214 | -0.0502 | -0.0298 |
|  | (-2.08) | (-0.12) | (0.82) | (-0.11) | (-0.16) |
| Sociodemographic characteristics |  |  |  |  |  |
| Male (vs. female) | -0.444 | 1.614^*^ | 0.390 | -1.197^**^ | -0.493^**^ |
|  | (-0.58) | (2.17) | (1.59) | (-2.82) | (-2.80) |
| Race-ethnicity (ref: White) |  |  |  |  |  |
| Mixed ethnicity | 1.624 | 2.229 | -1.202 | -0.720 | -0.276 |
|  | (0.65) | (0.91) | (-1.15) | (-0.52) | (-0.47) |
|  |  |  |  |  |  |
| Asian | -0.638 | 2.597^**^ | -0.496 | -2.273^***^ | -0.873^***^ |
|  | (-0.64) | (2.64) | (-1.71) | (-4.10) | (-3.36) |
|  |  |  |  |  |  |
| Black | -1.173 | 0.824 | -0.819 | -0.191 | 0.138 |
|  | (-0.54) | (0.39) | (-1.24) | (-0.16) | (0.30) |
|  |  |  |  |  |  |
| Other ethnicity | -2.195 | 2.718 | -0.874 | -0.430 | -0.0226 |
|  | (-0.78) | (0.98) | (-0.81) | (-0.27) | (-0.04) |
| Education around age 25 (ref: high) |  |  |  |  |  |
| Low Education Level | -1.561 | -1.061 | 0.195 | 0.0684 | 0.112 |
|  | (-0.83) | (-0.58) | (0.46) | (0.07) | (0.28) |
|  |  |  |  |  |  |
| Medium Education Level | -0.213 | -0.663 | -0.0571 | 0.493 | 0.184 |
|  | (-0.31) | (-1.00) | (-0.28) | (1.30) | (1.20) |
|  |  |  |  |  |  |
| Not-partnered around age 25 (vs. partnered) | -1.867^**^ | -1.241 | 0.479^*^ | 0.601 | 0.246 |
|  | (-2.78) | (-1.88) | (2.56) | (1.61) | (1.67) |
| Primary occupation between ages 25-34 (ref: professional/managerial) |  |  |  |  |  |
| Occupation missing (primarily due to not working) | -6.722^**^ | -1.666 | 1.580^**^ | 0.825 | 0.171 |
|  | (-3.25) | (-0.82) | (2.75) | (0.71) | (0.39) |
|  |  |  |  |  |  |
| Other occupations | -0.980 | 0.0407 | 0.353 | 0.196 | 0.0881 |
|  | (-1.01) | (0.04) | (1.17) | (0.36) | (0.40) |
|  |  |  |  |  |  |
| Clerks/Service- and Sales-related Workers | -0.587 | -0.400 | 0.526^*^ | 0.472 | -0.00774 |
|  | (-0.77) | (-0.53) | (2.15) | (1.11) | (-0.04) |
| Primary weekly working hours between ages 25-34 (ref: full-time) |  |  |  |  |  |
| Weekly hours missing (primarily due to not working) | -2.063 | -1.571 | 0.308 | 1.448 | 0.524 |
|  | (-1.23) | (-0.95) | (0.62) | (1.54) | (1.49) |
|  |  |  |  |  |  |
| Equal share of part- and full-time | -1.096 | 1.278 | 0.346 | -0.246 | 0.310 |
|  | (-0.62) | (0.74) | (0.66) | (-0.25) | (0.84) |
|  |  |  |  |  |  |
| Part-time (1-34 hrs/wk) | -2.358^**^ | -0.918 | 0.567^*^ | -0.607 | -0.0680 |
|  | (-2.93) | (-1.16) | (2.23) | (-1.35) | (-0.38) |
|  |  |  |  |  |  |
| Constant | 53.32^***^ | 49.69^***^ | -2.889^***^ | 12.06^***^ | -0.954^***^ |
|  | (71.69) | (68.06) | (-10.80) | (29.03) | (-5.73) |
| Observations | 1202 | 1202 | 1219 | 1204 | 1204 |

*Note.* NW: not working; ST: standard daytime hours; other NST: non-daytime hours other than evenings/nights (e.g., weekends, irregular). *t*-statistics in parentheses.

^*^ *p* < 0.05, ^**^ *p* < 0.01, ^***^ *p* < 0.001.

| **UK: UKHLS Health Outcomes at Age 45** | **SF12-Physical Function** | **SF12-Mental Function** | **Self-assessed Poor/Fair Health** | **GHQ-12 Poor Subjective Well-Being** | **Psychological distress (GHQ-12>13)** |
| --- | --- | --- | --- | --- | --- |
| Work schedule patterns between ages 35-44 (ref: mainly ST) |  |  |  |  |  |
| Mainly NW to some ST+NST | -2.776^***^ | -2.250^**^ | 0.738^***^ | 0.941^*^ | -0.00622 |
|  | (-3.83) | (-2.95) | (3.96) | (2.25) | (-0.04) |
|  |  |  |  |  |  |
| Mainly Evenings to volatile | -0.793 | -1.255 | 0.216 | 0.754 | 0.0117 |
|  | (-1.05) | (-1.58) | (1.00) | (1.73) | (0.06) |
|  |  |  |  |  |  |
| Mainly other NST to ST+NST | -0.566 | -0.284 | -0.314 | 0.0778 | 0.0661 |
|  | (-0.90) | (-0.43) | (-1.54) | (0.21) | (0.44) |
|  |  |  |  |  |  |
| Mainly ST to missing | -1.178 | -1.758^*^ | 0.412^*^ | 0.905^*^ | 0.273 |
|  | (-1.81) | (-2.57) | (2.25) | (2.40) | (1.81) |
| Sociodemographic characteristics |  |  |  |  |  |
| Male (vs. female) | 0.0951 | 2.058^***^ | 0.00382 | -0.714^*^ | -0.298^*^ |
|  | (0.17) | (3.50) | (0.02) | (-2.21) | (-2.25) |
| Race-ethnicity (ref: White) |  |  |  |  |  |
| Mixed ethnicity | -0.150 | -4.081^*^ | 0.465 | 3.057^**^ | 0.591 |
|  | (-0.09) | (-2.27) | (1.12) | (3.08) | (1.61) |
|  |  |  |  |  |  |
| Asian | -2.852^***^ | 1.605^*^ | 0.0516 | -0.875^*^ | -0.236 |
|  | (-4.47) | (2.40) | (0.30) | (-2.38) | (-1.49) |
|  |  |  |  |  |  |
| Black | -2.315 | 3.847^**^ | -0.168 | -2.030^**^ | -0.398 |
|  | (-1.88) | (2.98) | (-0.51) | (-2.85) | (-1.26) |
|  |  |  |  |  |  |
| Other ethnicity | 1.655 | 1.326 | -0.360 | -0.232 | -0.309 |
|  | (0.74) | (0.56) | (-0.55) | (-0.18) | (-0.53) |
| Education around age 35 (ref: high) |  |  |  |  |  |
| Low Education Level | -2.837^*^ | -1.161 | 0.800^**^ | 0.858 | 0.597^*^ |
|  | (-2.29) | (-0.89) | (2.91) | (1.22) | (2.18) |
|  |  |  |  |  |  |
| Medium Education Level | -1.284^*^ | -1.025 | 0.440^**^ | 0.609^*^ | 0.301^*^ |
|  | (-2.57) | (-1.95) | (3.15) | (2.11) | (2.54) |
|  |  |  |  |  |  |
| Not-partnered around age 35 (vs. partnered) | -2.436^***^ | -0.0723 | 0.606^***^ | 0.0861 | 0.0721 |
|  | (-3.49) | (-0.10) | (3.62) | (0.21) | (0.45) |
| Primary occupation between ages 35-44 (ref: professional/managerial) |  |  |  |  |  |
| Occupation missing (primarily due to not working) | -6.095^***^ | -2.003 | 0.662^*^ | 1.153 | 0.259 |
|  | (-4.81) | (-1.51) | (2.14) | (1.59) | (0.90) |
|  |  |  |  |  |  |
| Other occupations | -2.147^**^ | 1.682^*^ | 0.267 | -1.471^***^ | -0.712^***^ |
|  | (-3.10) | (2.32) | (1.39) | (-3.68) | (-4.02) |
|  |  |  |  |  |  |
| Clerks/Service- and Sales-related Workers | -2.078^***^ | 0.785 | 0.334^*^ | -0.606 | -0.344^*^ |
|  | (-3.57) | (1.28) | (2.01) | (-1.80) | (-2.49) |
| Primary weekly working hours between ages 35-44 (ref: full-time) |  |  |  |  |  |
| Weekly hours missing (primarily due to not working) | 1.125 | -0.222 | 0.103 | 0.205 | 0.110 |
|  | (1.31) | (-0.25) | (0.45) | (0.42) | (0.54) |
|  |  |  |  |  |  |
| Equal share of part- and full-time | -0.0567 | 1.084 | 0.217 | -0.510 | -0.124 |
|  | (-0.03) | (0.57) | (0.47) | (-0.49) | (-0.28) |
|  |  |  |  |  |  |
| Part-time (1-34 hrs/wk) | 0.0774 | 0.722 | -0.0187 | -0.258 | -0.00549 |
|  | (0.13) | (1.20) | (-0.11) | (-0.78) | (-0.04) |
|  |  |  |  |  |  |
| Constant | 52.43^***^ | 48.78^***^ | -2.335^***^ | 12.09^***^ | -0.918^***^ |
|  | (92.19) | (81.68) | (-13.42) | (36.87) | (-6.89) |
| Observations | 2073 | 2073 | 2098 | 2085 | 2085 |

*Note.* NW: not working; ST: standard daytime hours; other NST: non-daytime hours other than evenings/nights (e.g., weekends, irregular). *t*-statistics in parentheses.

^*^ *p* < 0.05, ^**^ *p* < 0.01, ^***^ *p* < 0.001.

a

| **UK: UKHLS Health Outcomes t Age 55** | **SF12-Physical Function** | **SF12-Mental Function** | **Self-assessed Poor/Fair Health** | **GHQ-12 Poor Subjective Well-Being** | **Psychological distress (GHQ-12>13)** |
| --- | --- | --- | --- | --- | --- |
| Work schedule patterns between ages 45-54 (ref: mainly ST) |  |  |  |  |  |
| Mainly NW | -7.572^***^ | -4.883^**^ | 1.247^***^ | 3.555^***^ | 0.796^*^ |
|  | (-4.89) | (-3.04) | (3.76) | (4.10) | (2.44) |
|  |  |  |  |  |  |
| Mainly ST + some NST | -0.864 | -0.215 | 0.119 | -0.0118 | 0.174 |
|  | (-1.42) | (-0.34) | (0.72) | (-0.03) | (1.21) |
|  |  |  |  |  |  |
| Mainly other NST + Evenings | -1.425 | 0.473 | 0.109 | -0.642 | -0.143 |
|  | (-1.95) | (0.62) | (0.55) | (-1.57) | (-0.78) |
| Sociodemographic characteristics |  |  |  |  |  |
| Male (vs. female) | -0.727 | 0.478 | 0.276 | -0.101 | -0.192 |
|  | (-1.26) | (0.80) | (1.81) | (-0.32) | (-1.40) |
| Race-ethnicity (ref: White) |  |  |  |  |  |
| Mixed ethnicity | -0.337 | 0.810 | -0.0370 | -0.964 | -0.351 |
|  | (-0.13) | (0.31) | (-0.06) | (-0.67) | (-0.53) |
|  |  |  |  |  |  |
| Asian | -2.211^*^ | -1.593 | 0.178 | 0.396 | 0.117 |
|  | (-2.43) | (-1.69) | (0.84) | (0.79) | (0.57) |
|  |  |  |  |  |  |
| Black | -3.085^*^ | 1.432 | 0.0813 | -0.652 | 0.0125 |
|  | (-2.50) | (1.12) | (0.26) | (-0.96) | (0.04) |
|  |  |  |  |  |  |
| Other ethnicity | -4.136 | -6.462^*^ | 0.934 | 5.152^***^ | 1.300^*^ |
|  | (-1.55) | (-2.33) | (1.59) | (3.44) | (2.34) |
| Education around age 45 (ref: high) |  |  |  |  |  |
| Low Education Level | -2.728^*^ | -1.839 | 0.350 | 0.285 | -0.100 |
|  | (-2.09) | (-1.36) | (1.18) | (0.39) | (-0.34) |
|  |  |  |  |  |  |
| Medium Education Level | -1.203^*^ | -0.276 | 0.120 | 0.0354 | -0.0755 |
|  | (-2.05) | (-0.45) | (0.78) | (0.11) | (-0.54) |
|  |  |  |  |  |  |
| Not-partnered around age 45 (vs. partnered) | -0.605 | -0.964 | 0.252 | 0.646 | 0.208 |
|  | (-0.79) | (-1.22) | (1.32) | (1.51) | (1.21) |
| Primary occupation between ages 45-54 (ref: professional/managerial) |  |  |  |  |  |
| Occupation missing (primarily due to not working) | -6.310^**^ | -4.031^*^ | 1.565^***^ | 0.495 | 0.621 |
|  | (-3.29) | (-2.03) | (3.48) | (0.46) | (1.48) |
|  |  |  |  |  |  |
| Other occupations | -2.001^*^ | 0.255 | 0.362 | 0.0535 | 0.158 |
|  | (-2.50) | (0.31) | (1.73) | (0.12) | (0.81) |
|  |  |  |  |  |  |
| Clerks/Service- and Sales-related Workers | -1.475^*^ | -0.534 | 0.432^*^ | 0.522 | 0.309 |
|  | (-2.17) | (-0.76) | (2.38) | (1.38) | (1.95) |
| Primary weekly working hours between ages 45-54 (ref: full-time) |  |  |  |  |  |
| Weekly hours missing (primarily due to not working) | -0.340 | -0.116 | -0.143 | -0.811 | -0.452 |
|  | (-0.25) | (-0.08) | (-0.33) | (-1.04) | (-1.34) |
|  |  |  |  |  |  |
| Equal share of part- and full-time | -0.100 | -2.892 | 0.744 | -0.754 | 0.191 |
|  | (-0.03) | (-0.97) | (1.00) | (-0.47) | (0.29) |
|  |  |  |  |  |  |
| Part-time (1-34 hrs/wk) | -2.477^*^ | -0.705 | 0.532 | -0.261 | -0.235 |
|  | (-2.14) | (-0.59) | (1.50) | (-0.40) | (-0.86) |
|  |  |  |  |  |  |
| Constant | 54.42^***^ | 50.89^***^ | -2.627^***^ | 11.62^***^ | -1.046^***^ |
|  | (42.61) | (38.47) | (-6.76) | (16.27) | (-3.48) |
| Observations | 1578 | 1578 | 1604 | 1588 | 1588 |

*Note.* NW: not working; ST: standard daytime hours; other NST: non-daytime hours other than evenings/nights (e.g., weekends, irregular). *t*-statistics in parentheses.

^*^ *p* < 0.05, ^**^ *p* < 0.01, ^***^ *p* < 0.001.

| **US: NLSY79 Health Outcomes at Age 40** | **SF12-Physical Function** | **SF12-Mental Function** | **Self-assessed Poor/Fair Health** | **CES-D Score** | **At risk of clinical depression (CES-D>=8)** |
| --- | --- | --- | --- | --- | --- |
| Work schedule patterns between ages 25-34 (ref: mainly ST) |  |  |  |  |  |
| Volatile to mainly NW | -2.567^***^ | -1.551^***^ | 0.736^***^ | 1.007^***^ | 0.441^***^ |
|  | (-7.30) | (-4.30) | (6.16) | (5.52) | (3.78) |
|  |  |  |  |  |  |
| Volatile to mainly other NST | -0.754^*^ | -0.112 | 0.422^***^ | 0.388^*^ | 0.223 |
|  | (-2.30) | (-0.33) | (3.34) | (2.29) | (1.80) |
|  |  |  |  |  |  |
| Volatile | -0.553^*^ | -0.616^*^ | 0.290^**^ | 0.492^***^ | 0.268^**^ |
|  | (-2.06) | (-2.24) | (2.81) | (3.54) | (2.73) |
| Sociodemographic characteristics |  |  |  |  |  |
| Male (vs. female) | 1.067^***^ | 2.209^***^ | -0.252^*^ | -1.182^***^ | -0.692^***^ |
|  | (4.22) | (8.51) | (-2.56) | (-9.01) | (-7.07) |
| Race-ethnicity (ref: non-Hispanic White) |  |  |  |  |  |
| Non-Hispanic Black | -0.476 | 0.459 | 0.326^***^ | 0.306^*^ | 0.131 |
|  | (-1.86) | (1.75) | (3.40) | (2.31) | (1.43) |
|  |  |  |  |  |  |
| Hispanic | 0.166 | 0.614^*^ | 0.310^**^ | -0.180 | -0.0276 |
|  | (0.59) | (2.14) | (3.00) | (-1.24) | (-0.27) |
|  |  |  |  |  |  |
| Other races | -1.115 | -1.176 | 0.295 | 0.551 | -0.141 |
|  | (-1.10) | (-1.14) | (0.81) | (1.05) | (-0.36) |
| Education by age 25 (ref: high) |  |  |  |  |  |
| Low Education Level | -3.838^***^ | -2.169^***^ | 1.679^***^ | 1.994^***^ | 1.248^***^ |
|  | (-8.96) | (-4.93) | (7.64) | (8.98) | (6.60) |
|  |  |  |  |  |  |
| Medium Education Level | -1.967^***^ | -0.592 | 0.963^***^ | 0.848^***^ | 0.721^***^ |
|  | (-5.57) | (-1.63) | (4.61) | (4.64) | (4.12) |
|  |  |  |  |  |  |
| Not-partnered around age 25 (vs. partnered) | -0.205 | -0.719^**^ | 0.269^**^ | 0.407^***^ | 0.177^*^ |
|  | (-0.95) | (-3.24) | (3.28) | (3.63) | (2.24) |
| Primary occupation between ages 25-34 (ref: professional/managerial) |  |  |  |  |  |
| Occupation missing (primarily due to not working) | -1.541 | 0.341 | 1.126^**^ | 0.884 | 0.389 |
|  | (-1.31) | (0.28) | (2.73) | (1.46) | (1.00) |
|  |  |  |  |  |  |
| Other Occupations | -0.700^*^ | -0.581 | 0.515^**^ | 0.602^**^ | 0.569^***^ |
|  | (-1.96) | (-1.59) | (3.18) | (3.26) | (3.80) |
|  |  |  |  |  |  |
| Service- or sales-related workers | -0.534 | -0.174 | 0.483^**^ | 0.318 | 0.331^*^ |
|  | (-1.64) | (-0.52) | (3.14) | (1.89) | (2.38) |
| Primary weekly working hours between ages 25-34 (ref: full-time) |  |  |  |  |  |
| Weekly hours missing (primarily due to not working) | 0.771 | -1.039 | -0.645 | -0.315 | 0.102 |
|  | (0.70) | (-0.92) | (-1.68) | (-0.55) | (0.29) |
|  |  |  |  |  |  |
| Equal share of part- and full-time | -0.264 | -0.560 | -0.0842 | 0.231 | 0.246 |
|  | (-0.46) | (-0.95) | (-0.42) | (0.77) | (1.37) |
|  |  |  |  |  |  |
| Part-time (1-34 hrs/wk) | 0.437 | -0.332 | -0.133 | 0.0900 | 0.0408 |
|  | (1.29) | (-0.95) | (-1.07) | (0.51) | (0.36) |
|  |  |  |  |  |  |
| Constant | 54.84^***^ | 53.62^***^ | -3.832^***^ | 2.002^***^ | -2.983^***^ |
|  | (156.63) | (149.30) | (-17.77) | (11.05) | (-16.84) |
| Observations | 5888 | 5888 | 5919 | 5864 | 5864 |

*Note.* NW: not working; ST: standard daytime hours; other NST: non-daytime hours other than evenings/nights (e.g., weekends, irregular). *t*-statistics in parentheses.

^*^ *p* < 0.05, ^**^ *p* < 0.01, ^***^ *p* < 0.001.

| **US: NLSY79 Health Outcomes at Age 50** | **SF12-Physical Function** | **SF12-Mental Function** | **Self-assessed Poor/Fair Health** | **CES-D score** | **At risk of clinical depression (CES-D>=8)** |
| --- | --- | --- | --- | --- | --- |
| Work schedule patterns between ages 35-44 (ref: ST only) |  |  |  |  |  |
| Mainly NW to some ST | -2.081^***^ | -1.604^***^ | 0.427^***^ | 0.783^***^ | 0.321^*^ |
|  | (-4.12) | (-3.61) | (3.36) | (3.49) | (2.39) |
|  |  |  |  |  |  |
| ST + NST | -1.037^***^ | -0.676^*^ | 0.193^*^ | 0.445^***^ | 0.288^**^ |
|  | (-3.42) | (-2.54) | (2.26) | (3.30) | (3.19) |
|  |  |  |  |  |  |
| Mainly other NST | -0.638 | -0.438 | 0.193 | 0.404 | 0.189 |
|  | (-1.34) | (-1.04) | (1.43) | (1.91) | (1.34) |
| Sociodemographic characteristics |  |  |  |  |  |
| Male (vs. female) | 1.671^***^ | 2.081^***^ | -0.213^*^ | -1.319^***^ | -0.696^***^ |
|  | (5.36) | (7.58) | (-2.43) | (-9.51) | (-7.36) |
| Race-ethnicity (ref: non-Hispanic White) |  |  |  |  |  |
| Non-Hispanic Black | -0.520 | 1.095^***^ | 0.220^*^ | -0.292^*^ | -0.234^*^ |
|  | (-1.59) | (3.80) | (2.53) | (-2.01) | (-2.53) |
|  |  |  |  |  |  |
| Hispanic | 0.514 | 1.013^**^ | 0.104 | -0.623^***^ | -0.328^**^ |
|  | (1.46) | (3.26) | (1.09) | (-3.97) | (-3.21) |
|  |  |  |  |  |  |
| Other races | 0.633 | 0.0803 | 0.137 | -0.443 | -0.126 |
|  | (0.50) | (0.07) | (0.39) | (-0.79) | (-0.35) |
| Education by age 35 (ref: high) |  |  |  |  |  |
| Low Education Level | -4.777^***^ | -1.658^***^ | 1.486^***^ | 1.751^***^ | 1.062^***^ |
|  | (-9.63) | (-3.79) | (9.52) | (7.93) | (7.07) |
|  |  |  |  |  |  |
| Medium Education Level | -2.998^***^ | -0.0483 | 0.962^***^ | 0.677^***^ | 0.625^***^ |
|  | (-8.06) | (-0.15) | (6.93) | (4.09) | (4.89) |
|  |  |  |  |  |  |
| Not-partnered around age 25 (vs. partnered) | -1.279^***^ | -1.375^***^ | 0.380^***^ | 0.798^***^ | 0.442^***^ |
|  | (-4.22) | (-5.16) | (4.90) | (5.92) | (5.40) |
| Primary occupation between ages 35-44 (ref: professional/managerial) |  |  |  |  |  |
| Occupation missing (primarily due to not working) | 0.779 | -1.490 | 0.134 | 0.539 | 0.224 |
|  | (0.55) | (-1.19) | (0.42) | (0.85) | (0.67) |
|  |  |  |  |  |  |
| Other Occupations | -1.587^***^ | -0.866^*^ | 0.560^***^ | 0.691^***^ | 0.382^**^ |
|  | (-3.93) | (-2.43) | (4.78) | (3.83) | (3.16) |
|  |  |  |  |  |  |
| Service- or sales-related workers | -0.877^*^ | -0.417 | 0.343^**^ | 0.301 | 0.137 |
|  | (-2.47) | (-1.33) | (3.19) | (1.90) | (1.29) |
| Primary weekly working hours between ages 35-44 (ref: full-time) |  |  |  |  |  |
| Weekly hours missing (primarily due to not working) | -7.299^***^ | -1.561 | 0.919^***^ | 0.766 | 0.451 |
|  | (-5.76) | (-1.40) | (3.29) | (1.36) | (1.55) |
|  |  |  |  |  |  |
| Equal share of part- and full-time | -1.208 | -1.029 | 0.185 | 0.0793 | 0.0331 |
|  | (-1.45) | (-1.40) | (0.91) | (0.22) | (0.16) |
|  |  |  |  |  |  |
| Part-time (1-34 hrs/wk) | -0.343 | -0.561 | 0.196 | 0.286 | 0.101 |
|  | (-0.79) | (-1.47) | (1.71) | (1.49) | (0.89) |
|  |  |  |  |  |  |
| Constant | 53.74^***^ | 53.53^***^ | -3.101^***^ | 2.879^***^ | -2.405^***^ |
|  | (138.85) | (157.01) | (-21.06) | (16.71) | (-18.03) |
| Observations | 5572 | 5572 | 5619 | 5575 | 5575 |

*Note.* NW: not working; ST: standard daytime hours; other NST: non-daytime hours other than evenings/nights (e.g., weekends, irregular). *t*-statistics in parentheses.

^*^ *p* < 0.05, ^**^ *p* < 0.01, ^***^ *p* < 0.001.

| **US: NLSY79 Health Outcomes at Age 60** | **SF12-Physical Function** | **SF12-Mental Function** | **Self-assessed Poor/Fair Health** | **CES-D score** | **At risk of clinical depression (CES-D>=8)** |
| --- | --- | --- | --- | --- | --- |
| Work schedule patterns between ages 45-54 (ref: ST only) |  |  |  |  |  |
| Mainly NW | -3.631^***^ | -0.559 | 0.180 | 0.223 | 0.187 |
|  | (-3.96) | (-0.78) | (0.91) | (0.57) | (0.77) |
|  |  |  |  |  |  |
| ST+NST to mainly ST | -1.209^*^ | -0.314 | 0.164 | 0.273 | 0.263 |
|  | (-2.04) | (-0.68) | (1.20) | (1.09) | (1.50) |
|  |  |  |  |  |  |
| ST to other NST | -2.058^**^ | -1.021 | 0.295 | 0.887^**^ | 0.561^**^ |
|  | (-2.58) | (-1.64) | (1.63) | (2.71) | (2.60) |
|  |  |  |  |  |  |
| Other NST to ST | -0.817 | -0.222 | 0.0248 | 0.397 | 0.374 |
|  | (-1.00) | (-0.35) | (0.12) | (1.19) | (1.62) |
|  |  |  |  |  |  |
| Mainly Evenings/Nights | -1.319 | -1.395^**^ | 0.313^*^ | 0.675^*^ | 0.382 |
|  | (-1.93) | (-2.63) | (2.08) | (2.36) | (1.93) |
|  |  |  |  |  |  |
| Mainly other NST | -2.655^***^ | -0.614 | 0.308 | 0.808^**^ | 0.708^***^ |
|  | (-3.88) | (-1.15) | (1.95) | (2.78) | (3.74) |
| Sociodemographic characteristics |  |  |  |  |  |
| Male (vs. female) | 1.271^**^ | 1.477^***^ | -0.175 | -1.273^***^ | -0.820^***^ |
|  | (2.74) | (4.09) | (-1.57) | (-6.57) | (-5.52) |
| Race-ethnicity (ref: non-Hispanic White) |  |  |  |  |  |
| Non-Hispanic Black | -1.760^***^ | 0.686 | 0.278^*^ | -0.335 | -0.145 |
|  | (-3.56) | (1.78) | (2.50) | (-1.61) | (-1.03) |
|  |  |  |  |  |  |
| Hispanic | 0.940 | 1.198^**^ | 0.0378 | -0.469^*^ | -0.150 |
|  | (1.77) | (2.90) | (0.31) | (-2.10) | (-0.97) |
|  |  |  |  |  |  |
| Other races | -0.686 | 0.895 | -0.253 | -0.370 | -0.704 |
|  | (-0.33) | (0.56) | (-0.45) | (-0.43) | (-0.93) |
| Education by age 45 (ref: high) |  |  |  |  |  |
| Low Education Level | -6.475^***^ | -2.193^***^ | 1.539^***^ | 1.520^***^ | 0.768^***^ |
|  | (-8.28) | (-3.60) | (8.54) | (4.66) | (3.52) |
|  |  |  |  |  |  |
| Medium Education Level | -3.010^***^ | -0.584 | 0.756^***^ | 0.464^*^ | 0.353^*^ |
|  | (-5.83) | (-1.45) | (5.35) | (2.16) | (2.13) |
|  |  |  |  |  |  |
| Not-partnered around age 45 (vs. partnered) | -0.747 | -0.728^*^ | 0.201^*^ | 0.246 | 0.205 |
|  | (-1.69) | (-2.12) | (2.04) | (1.34) | (1.67) |
| Primary occupation between ages 45-54 (ref: professional/managerial) |  |  |  |  |  |
| Occupation missing (primarily due to not working) | -0.489 | -0.416 | 0.106 | 0.414 | 0.543 |
|  | (-0.30) | (-0.33) | (0.31) | (0.60) | (1.35) |
|  |  |  |  |  |  |
| Other Occupations | -1.218 | -0.507 | 0.254 | 0.670^*^ | 0.498^*^ |
|  | (-1.89) | (-1.01) | (1.65) | (2.49) | (2.49) |
|  |  |  |  |  |  |
| Service- or sales-related workers | -0.542 | -0.753 | 0.222 | 0.422 | 0.270 |
|  | (-1.04) | (-1.86) | (1.72) | (1.94) | (1.69) |
| Primary weekly working hours between ages 45-54 (ref: full-time) |  |  |  |  |  |
| Weekly hours missing (primarily due to not working) | -7.501^***^ | -3.344^**^ | 1.115^***^ | 2.047^**^ | 0.814^*^ |
|  | (-4.60) | (-2.64) | (3.35) | (2.95) | (2.08) |
|  |  |  |  |  |  |
| Equal share of part- and full-time | -4.990^***^ | -2.592^*^ | 1.017^***^ | 2.153^***^ | 0.904^**^ |
|  | (-3.48) | (-2.32) | (3.72) | (3.54) | (2.80) |
|  |  |  |  |  |  |
| Part-time (1-34 hrs/wk) | -0.772 | -0.922 | 0.111 | 0.353 | 0.0960 |
|  | (-1.18) | (-1.81) | (0.75) | (1.28) | (0.54) |
|  |  |  |  |  |  |
| Constant | 51.74^***^ | 54.29^***^ | -2.489^***^ | 2.801^***^ | -2.385^***^ |
|  | (97.35) | (131.28) | (-16.62) | (12.68) | (-13.62) |
| Observations | 3032 | 3032 | 3065 | 2528 | 2528 |

*Note.* NW: not working; ST: standard daytime hours; other NST: non-daytime hours other than evenings/nights (e.g., weekends, irregular). *t*-statistics in parentheses.

^*^ *p* < 0.05, ^**^ *p* < 0.01, ^***^ *p* < 0.001.
