# Supplementary material for: Longitudinal employment patterns and parental health: A cross-country look
Source: PLoS One. 2026 Jun 5;21(6):e0350945. doi: 10.1371/journal.pone.0350945 (PMC13240889; doi:10.1371/journal.pone.0350945)
Supplement: S3 Fig — (DOCX) [file pone.0350945.s007.docx]

**S3. Fig. Sequence Analysis Distribution Plot for Work Arrangement Among Parents aged 45-54**

**S3.1. Australia**





**S3.2. Germany**





**S3.3. The United Kingdom**





**S3.4. The United States**
