# Supplementary material for: Longitudinal employment patterns and parental health: A cross-country look
Source: PLoS One. 2026 Jun 5;21(6):e0350945. doi: 10.1371/journal.pone.0350945 (PMC13240889; doi:10.1371/journal.pone.0350945)
Supplement: S1 File — (DOCX) [file pone.0350945.s008.docx]

**S1. File.** Data Details

**Australia: Household, Income and Labour Dynamics in Australia (HILDA).**

The HILDA Project was initiated and is funded by the Australian Commonwealth Department of Social Services (DSS) and is managed by the Melbourne Institute of Applied Economic and Social Research (Melbourne Institute). HILDA is a household-based panel study of a nationally representative sample of Australians, surveying more than 17,000 individuals and 7,000 households since 2001. The survey collects detailed information about individuals aged 15 and over on current employment, job characteristics, income, work schedule, employment histories, histories of individuals’ sociodemographic characteristics (e.g., education, marriage, sex, family structure), and health measures (e.g., physical, mental health, and psychological well-being). Attrition rates are low, with more than 90% of respondents in any wave responding in the next wave. For a detailed discussion of the survey design and user manuals, technical papers and related publications, see https://melbourneinstitute.unimelb.edu.au/hilda. HILDA publicly available data are only available upon approval from the Australian Data Custodian (ADA Data Archive). See more here: <https://dataverse.ada.edu.au/dataverse.xhtml?alias=hilda&utm_source>. As a legal requirement, we are not allowed to upload any data. However, the data is publicly available upon approval from the Melbourne Institute for HILDA data.

**Germany: The German Socio-Economic Panel (SOEP)**

SOEP is a nationally representative, longitudinal survey of private households in Germany, conducted annually since 1984. To maintain representativeness and address issues such as panel attrition, SOEP has regularly added refreshment samples. These include not only general population samples (in 1998, 2006, 2012, 2020) but also targeted groups, such as East Germans after reunification (sampled in 1990), recent immigrants (sampled in 1994), and refugees (sampled in 2016 and 2020). As of recent waves, SOEP includes around 30,000 respondents from more than 15,000 households. Overall, attrition rates are low, with about 98% of respondents from one wave also participating in the subsequent wave. SOEP collects rich, individual- and household-level data on a wide range of topics, including income, employment (e.g., work hours, work schedules), education, health (e.g., physical and mental health), and family dynamics on individuals aged 16 and older. SOEP is publicly available from the German Institute for Economic Research (DIW, https://www.diw.de/en/soep, DOI: 10.5684/soep.core.v41eu). As with the HILDA data, individual researchers must apply to use the publicly available SOEP data. Please see details here: <https://www.diw.de/en/diw_01.c.601584.en/data_access.html>

**UK: Understanding Society (UKHLS)**

UKHLS is a nationally representative longitudinal household panel study conducted by the Institute for Social and Economic Research at the University of Essex and designed to reflect the full diversity of the UK population. Participants are drawn from all four UK nations and across a wide range of age groups, educational backgrounds, and social strata. Launched in 2009, UKHLS began with a sample of approximately 40,000 households, including around 8,000 retained from the earlier British Household Panel Survey (BHPS), which ran from 1991 to 2009. The inclusion of BHPS respondents allows for the analysis of individual and family trajectories spanning more than three decades. The sample includes approximately 10,000 individuals per birth cohort per decade from the 1940s onward, as well as around 17,000 children born into the study since 2000. The survey collects detailed information on sociodemographic characteristics, employment conditions, physical and mental health, and household composition, with additional linkages to administrative records and geographic identifiers. With a re-interview rate of approximately 90%, the study maintains a high level of panel stability. However, in contrast to other longitudinal datasets with calendar-year-based fieldwork, each UKHLS wave is conducted over an intended 24-month period, with some interviews completed slightly beyond that window. As a result, both the sequence analysis and the regression models using UKHLS data are aligned on a wave-based timeline. Although information on work schedules is collected every second wave, the availability of consistent yearly data on job characteristics, health outcomes, and parenthood and partnership status makes UKHLS well-suited for the analyses presented in this study. Individual researchers need to apply for the use of publicly-available UKHLS, see details here: https://www.understandingsociety.ac.uk/help/new-user-pathway/

**US: National Longitudinal Survey of Youth-1979 (NLSY79)**

NLSY79 was conducted by the U.S. Department of Labor, consisting of a large nationally representative sample of 12,686 young men and women ages 14 to 22 when first interviewed in 1979, with annual interviews until 1994 and biennially thereafter. During early years due to budget constraints, NLSY79 discontinued two oversamples: an oversampling of military youth was discontinued in 1984 (n = 1280) and an oversampling of non-Black non-Hispanic disadvantaged youths was discontinued in 1990 (n = 1,643). This study excludes these oversampled participants, resulting in a total of 9,763 respondents as the starting point. Despite the NLSY79 team following up with the same participants for about 40 years, the response rates have been remarkably high, from as high as 96% in the early years of the survey to about 65% in the most recent data collection year 2022 (National Longitudinal Survey [NLS], n.d.). The sample retained in the most recent year, 2020, was about 6,300 (NLS, n.d.). This nationally representative sample of young adults in 1979 contains rich data, including histories of individuals’ sociodemographic characteristics (e.g., education, marriage, family structure, income), annual work schedule and employment histories, and a set of physical and psychological well-being metrics. The NLSY79 thus remains the only study with a nationally representative sample that includes longitudinal information on work schedules over one’s working years. The health outcome variables in the NLSY79 were collected through their health modules at ages 40, 50, and 60. The NLSY79 data can be accessed via NLS Investigator portal after registering with the system. Please see details on the following two links: (1) Data overview: <https://www.bls.gov/nls/nlsy79.htm> (2) NLS Investigator portal: <https://www.nlsinfo.org/investigator/pages/login>

National Longitudinal Surveys (NLS). (n.d.). Naitonal Longitudinal Survey of Youth 1979: Retentions & reasons for nonterview. Retrieve on July 2, 2025 [https://www.nlsinfo.org/content/cohorts/nlsy79/intro-to-the-sample/retention-reasons-noninterview#:~:text=response%20rate,be%20alive%20is%2074.4%20percent](https://www.nlsinfo.org/content/cohorts/nlsy79/intro-to-the-sample/retention-reasons-noninterview#:~:text=response%20rate,be%20alive%20is%2074.4%20percent)
