# Supplementary material for: Longitudinal employment patterns and parental health: A cross-country look
Source: PLoS One. 2026 Jun 5;21(6):e0350945. doi: 10.1371/journal.pone.0350945 (PMC13240889; doi:10.1371/journal.pone.0350945)
Supplement: Attachment — Submitted filename: PONE-Response Memo March 2026.docx [file pone.0350945.s010.docx]

**PONE-D-25-46936: Longitudinal Employment Patterns and Parental Health: A Cross-Country Look**

**RESPONSE TO THE EDITOR:**

We thank the reviewers for their very helpful and constructive comments in strengthening this manuscript. We have revised the paper based on the comments raised by the reviewers and have responded to each point in the responses below. **The major changes are in the tracked changes.**

**RESPONSE to Editorial Comment on “Journal Requirement”**

Journal Requirements:

When submitting your revision, we need you to address these additional requirements.

1.Please ensure that your manuscript meets PLOS ONE's style requirements, including those for file naming. The PLOS ONE style templates can be found at

[https://journals.plos.org/plosone/s/file?id=wjVg/PLOSOne_formatting_sample_main_body.pdf](https://urldefense.proofpoint.com/v2/url?u=https-3A__journals.plos.org_plosone_s_file-3Fid-3DwjVg_PLOSOne-5Fformatting-5Fsample-5Fmain-5Fbody.pdf&d=DwQFaQ&c=slrrB7dE8n7gBJbeO0g-IQ&r=ltiwpPv12BiRwl__ZHEpxg&m=-s4l1uToD05bLLqO8L7BQyYDj1O-34_LtHk1dunT7lfEVacZD9HECtIaB2UOPDcP&s=dbyusCLJwC0yHENqrA0odXS35JSbANFnub2ADU4smIM&e=) and

[https://journals.plos.org/plosone/s/file?id=ba62/PLOSOne_formatting_sample_title_authors_affiliations.pdf](https://urldefense.proofpoint.com/v2/url?u=https-3A__track.editorialmanager.com_CL0_https-3A-252F-252Fjournals.plos.org-252Fplosone-252Fs-252Ffile-253Fid-3Dba62-252FPLOSOne-5Fformatting-5Fsample-5Ftitle-5Fauthors-5Faffiliations.pdf_1_010f019bf8cb63e0-2Dc714312f-2D1fc3-2D4525-2Dafd5-2Dfb5aefe7873c-2D000000_rrx2gaqC0-5FYQdUqZE7epuiH4Ef3IilzWmK2Lg-5FXlyrk-3D247&d=DwMFaQ&c=slrrB7dE8n7gBJbeO0g-IQ&r=ltiwpPv12BiRwl__ZHEpxg&m=-s4l1uToD05bLLqO8L7BQyYDj1O-34_LtHk1dunT7lfEVacZD9HECtIaB2UOPDcP&s=oZ8RjgPO6KGfHwf4-Fqs0v68_qHjP8ni63OKl70hDZY&e=)

[RESPONSE] Thank you for your careful checking of our manuscript. We have carefully followed the guidelines you provided to prepare our revised manuscript.

2. Thank you for uploading your study's underlying data set. Unfortunately, the repository you have noted in your Data Availability statement does not qualify as an acceptable data repository according to PLOS's standards.

At this time, please upload the minimal data set necessary to replicate your study's findings to a stable, public repository (such as figshare or Dryad) and provide us with the relevant URLs, DOIs, or accession numbers that may be used to access these data. For a list of recommended repositories and additional information on PLOS standards for data deposition, please see [https://journals.plos.org/plosone/s/recommended-repositories](https://urldefense.proofpoint.com/v2/url?u=https-3A__track.editorialmanager.com_CL0_https-3A-252F-252Fjournals.plos.org-252Fplosone-252Fs-252Frecommended-2Drepositories_1_010f019bf8cb63e0-2Dc714312f-2D1fc3-2D4525-2Dafd5-2Dfb5aefe7873c-2D000000_ryvVqpyEJoEZNndlQXnZ-2DrIOw5K5E2KfBwZZiiL79os-3D247&d=DwMFaQ&c=slrrB7dE8n7gBJbeO0g-IQ&r=ltiwpPv12BiRwl__ZHEpxg&m=-s4l1uToD05bLLqO8L7BQyYDj1O-34_LtHk1dunT7lfEVacZD9HECtIaB2UOPDcP&s=qxRoKzSl8lXRETnkOdSdCPOhm-oASHtimWW46OdnBkk&e=).

[RESPONSE] We provide the web links to individual data in our S1. File for accessing each of the four datasets we used. We have also provided statements about data from Australia, Germany, and the UK that require an individual researcher’s application for public data access, which we have obtained. For this reason, we do not upload the data for public access. To enhance transparency in our data analysis, we have provided detailed information on how we coded each variable across all four datasets shown in S1. Table.

3. Please include a copy of Table 1 and 2 which you refer to in your text on page 16 and 20.

[RESPONSE] We have now included the files for Tables 1 and 2 in our resubmission.

4. Please include captions for your Supporting Information files at the end of your manuscript, and update any in-text citations to match accordingly. Please see our Supporting Information guidelines for more information: [http://journals.plos.org/plosone/s/supporting-information](https://urldefense.proofpoint.com/v2/url?u=http-3A__track.editorialmanager.com_CL0_http-3A-252F-252Fjournals.plos.org-252Fplosone-252Fs-252Fsupporting-2Dinformation_1_010f019bf8cb63e0-2Dc714312f-2D1fc3-2D4525-2Dafd5-2Dfb5aefe7873c-2D000000_raRwOVi-2D3B535IGXf83714v3Va-5Fz86abDAcwfEBBK4w-3D247&d=DwMFaQ&c=slrrB7dE8n7gBJbeO0g-IQ&r=ltiwpPv12BiRwl__ZHEpxg&m=-s4l1uToD05bLLqO8L7BQyYDj1O-34_LtHk1dunT7lfEVacZD9HECtIaB2UOPDcP&s=JwDdo2FsL4bINc5zlBxWgYMSQCf_akC_UvRD4Ce-SUo&e=).

[RESPONSE] We now provide the captions of our supporting information at the end of the manuscript and ensure the reference to the supporting information in the text is accurately stated.

5. We note that there is identifying data in the Supporting Information file “S1 Table_PLOS supporting information.docx, S3 Table_PLOS supporting information.docx and S4 Table_PLOS supporting information.docx”. Due to the inclusion of these potentially identifying data, we have removed this file from your file inventory. Prior to sharing human research participant data, authors should consult with an ethics committee to ensure data are shared in accordance with participant consent and all applicable local laws

[RESPONSE] We have carefully examined each table to ensure no identifiable data is presented. We used publicly available data in aggregate form (e.g., means, large counts, regression results) to present those statistics. They are not identifiable by any means. We inquired about this issue with the editorial office and received a response confirming that our tables do not contain identifiable information (response dated February 4, 2026, from Kit Stokes).

**RESPONSE TO REVIEWER 1**

We thank this reviewer for the thoughtful comments and believe that the paper has been considerably improved. All comments are addressed below.

Areas for Improvement:

• Sample Sizes: As acknowledged by the authors, the sample sizes for Germany are relatively small, which may limit the power to detect statistically significant effects.

[RESPONSE] We thank this reviewer for this important point. We now further clarify in Lines 639-644 that the limited sample size in Germany is confined to respondents aged 35 and its associated limitations, whereas for the other two age groups (45 and 55), the sample sizes are larger and comparable to those in Australia and the United Kingdom.

• Voluntary vs. Involuntary Work Schedules: The analysis does not differentiate between voluntary and involuntary non-standard work schedules.

[RESPONSE] We thank this reviewer for raising this important issue. We discuss the implications of (in)voluntary work schedules for parental health in our Literature Review section (Lines 215-225), and we address this limitation in Lines 662-667, given the data at hand and its implications for our results.

• Social Determinants Discussion: Could be expanded on how social determinants influence health.

[RESPONSE] As suggested, we now add a discussion directly linking SDOH to health (Lines 101-104) and specify multiple aspects of nonstandard work (including hours, instability, volatility, and structural inequality) that closely align with the SDOH concept (Lines 104-111).

• Data Comparability: Although the authors do take steps to ensure data comparability, they can be further improved.

[RESPONSE] We thank the reviewer for this important point and we wholeheartedly agree with this point. We tried to harmonize as much as possible across the datasets; however, some variables were measured differently across countries, and we thus acknowledged this in the limitations section (Lines 644-651). In addition to the descriptions provided in the text (Lines 323-349), we address the data comparability issue by conducting separate regression analyses for each country rather than pooling the datasets. This analysis allows us to examine the relationship between work patterns and health outcomes within each country rather than across countries, thereby at least partially avoiding potential measurement noise from cross-country data harmonization. We now address this issue in our Limitations section (Lines 644-658).

Specific Comments and Suggestions:

• Introduction: To strengthen the introduction, the authors could briefly discuss the specific parental leave and childcare policies in the four countries under study, highlighting how they might relate to non-standard work arrangements.

[RESPONSE] We thank this reviewer for this helpful comment to strengthen the context introduction for this paper. We now add a statement to acknowledge the important role of institutional context including childcare policies and labor market policies in the Introduction section (Lines 44-56) and provide a detailed discussion concerning institutional context in the Literature Review section (Lines 130-189), with the intention to present the information in one place without repetition. We hope you agree with our organization. Given parental leave policy does not immediately or directly relate to our key independent variable, work schedules, we focus on childcare policy because the extant research has shown how the availability and affordability of childcare and the work schedule arrangement closely shape each other; we discuss this in the Literature Review section (Lines 215-242).

• Data and Methods: Justify the age range chosen. Explain how missing data was handled and if there were any limitations with the methodology.

[RESPONSE] We now provide details on the reasons for choosing the specific age range (Lines 324-328). In addition, we now specify the details of missing cases for each variable in our supporting information (S3 Table was unfortunately removed by the editorial office in the first submission), along with how we handled missing data for the independent and dependent variables (Lines 374-382).

• Results: Provide more specific examples of differences in health outcomes due to specific reasons.

[RESPONSE] We thank this reviewer for this helpful suggestion to make the results more relatable to readers. As suggested by Reviewer 2 as well, we now include discussions to qualify some of the results from Table 2, shown in Lines 462-477 and 548-566.

• Discussion: Focus on why parental work may carry long-lasting impacts on health, and how to deal with this issue.

[RESPONSE] We thank this reviewer for this valuable point. In addition to our discussion throughout the Literature Review section, which underscores the critical implications of parental work for the well-being of the next generation, we now add a discussion to underscore the critical roles of parental work and health when paired with important policies in achieving positive child outcomes, as shown in Lines 702-709.

Potential Typos and Misspellings:

• Page 7: "Jianghong Li, WZB Social Science Center Berlin, Germany; Curtin University and The Kids Institue, Western Australia" - "Institue" should be "Institute"
• Page 11: "sync of biological rthyms" - "rthyms" should be "rhythms"
• Page 14: "Perry-Jenkins et al" it is not consistent, some cases the last name is not hyphened. The name shoudl be the same.
• Page 15: "Specifically, the four countries examined in this study differ signifcantly in key" - "signifcantly" should be "significantly"
• Page 19: "Where the concept applies to the contry context" - "contry" should be "country"
• Page 21: "which with transforming one sequence into another"- It is "With" or "which"?

[RESPONSE] We thank this reviewer for this careful reading, our sincere apologies for our carelessness in spelling. We have now corrected these typos and carefully proofread our manuscript to ensure it is free of errors.

**RESPONSE TO REVIEWER 2**

We appreciate the reviewer's important and valuable comments and have addressed them as follows.

Relevance
While the paper addresses an important and timely topic, its broader relevance could be strengthened by more clearly articulating why parental wellbeing matters beyond individual health outcomes. The analysis convincingly documents associations between long-term work trajectories and parental health, but it stops short of situating these findings within a broader discussion of economic efficiency and social costs. In particular, the paper does not fully explore how deteriorations in parental health may affect labor supply, productivity, public health expenditures, or the intergenerational transmission of disadvantage. As a result, the relevance remains primarily descriptive, with the economic implications of the findings left largely implicit rather than central to the argument.

[RESPONSE] We thank this reviewer for this valuable feedback to strengthen the relevance of this empirical evidence to large scholarship. We now add a discussion in the Introduction section highlighting the links between work schedules and workers’ health, the economic and productivity costs to employers and society (Lines 21-28), and the significant implications for the intergenerational transmission of disadvantages (Lines 28-36). As our aim is to underscore the importance of parental work in transmitting (dis)advantages, we further situate parental health outcomes in the context of intergenerational transmission of disadvantage, as shown throughout our literature review and in Lines 702-709, as suggested by Reviewer 1.

In addition, to anchor our focus on cross-country analysis as well as addressing the important comments on institutional context from both you and Reviewer 1, we now place interpretations and discussions aligning more with the differences in institutional contexts concerning labor market and social protection provisions (e.g., working time regulation, childcare policy) throughout the paper, we hope you agree with our approach.

Method and Interpretation of Results
The authors are transparent about the subjective and structural aspects of the sequence analysis, including clustering decisions, treatment of missing states, and cross-country comparability constraints. These limitations are inherent to life-course sequence analysis using international panel data and are handled using standard, defensible practices, though the following points would benefit from revision:
Causality: The paper appropriately frames its results as associations rather than causal effects; however, given the academic and policy relevance of work schedules and labor-market arrangements, it is crucial to clarify these limitations from the outset. I encourage the authors to expand the discussion of causality by explicitly addressing: (i) why causal estimates would be particularly valuable in this context; (ii) what the reliance on associations implies for academic contribution and policy relevance; and (iii) how the life-course, trajectory-based approach, while not resolving endogeneity, helps mitigate some limitations of associational evidence by capturing cumulative exposure rather than point-in-time correlations.

[RESPONSE] We thank this reviewer for this helpful comment. We now add discussions in the front section of the paper (Lines 57-71) to specifically acknowledge (1) the importance of causality for policy-making, (2) this study’s inability to establish causality due to data at hand and ethical concerns, and (3) the advantage of longitudinal data together with sequence analysis in capturing long-term work patterns to mitigate measurement noise and address temporal sequence, and hence the importance of accumulating empirical evidence with better and sophisticated tools when experimental design is impossible/unethical.

Institutional context and cross-country interpretation: More detail on the legal and institutional context shaping labor-market flexibility and health protection across countries is needed to strengthen the interpretation of cross-country differences by more systematically linking observed patterns to institutional features such as working-time regulations, employment protection, schedule flexibility rights, and health-care system coverage.

[RESPONSE] As suggested, we now add discussions related to institutional contexts throughout our paper in the front section of the paper (Lines 44-56), the Literature Review section (Lines 130-189), the Results section (Lines 462-477, 548-566), and the Discussion section (Lines 616-636).

Origininality
The paper provides a solid discussion of the literature documenting associations between work instability and parental wellbeing; however, it is not clear how by providing evidence on associations the paper contribute academically and may have implication for policy. In particular, the reasons for the existing gap in cross-country evidence sufficiently discussed, whether due to data limitations or the complexity of comparing highly heterogeneous institutional contexts. Moreover, given that the paper does not report causal estimates, it would be useful to clarify how the analysis nevertheless adds value to the literature and complements the existing causal evidence. More specifically:
• Clarify why US-centric evidence is a limitation. More explicitly articulate why the dominance of US-based studies poses an external validity problem, particularly given differences in labor-market regulation, social protection, and health-care systems across countries.

[RESPONSE] We thank this reviewer for these valuable points regarding the originality and contribution of this association study. We now provide a more detailed discussion of the state of knowledge on the links between parental work schedules and parental health around the globe (Lines 252-266), followed by a discussion of the potential reasons for mixed findings shown in the extant knowledge (Lines 266-282) including why one country’s findings may not be applicable to other countries (Lines 278-286).

• Explain the source of the cross-country evidence gap: Clarify whether the lack of cross-country analyses in the literature primarily reflects data constraints or whether contextual heterogeneity has limited meaningful comparison, and explain how the paper navigates these challenges and add to the literature by dealing with data of countries with such differential features.

[RESPONSE] As suggested, we now add texts addressing how our cross-country study might add values to the body of scholarship on the links between work schedules and health in the Introduction section (Lines 44-71); the literature review section on country context (Lines 130-189); potential reasons for the mixed findings from the extant knowledge on the links between parental work schedules and parental health around the globe and how our study might build upon and extend beyond this body of scholarship (Lines 252-282); our methodology on data harmonization to navigate the challenges in cross-country analysis and results (Lines 322-349), and; our use of institutional lens to interpret the findings in the Results (Lines 462-477, 548-566) and Discussion section (Lines 616-621, 633-636) to underscore how a cross-country analysis with nationally longitudinal data could shed new light on the mixed picture shown in existing studies by carefully delineate the dynamic changes in work patterns. We hope these additions address this suggestion.

• Position the contribution relative to existing causal evidence. There is a growing body of work providing causal estimates of the effects of work instability/flexibility or even parental leave on parental and infant wellbeing that is not fully discussed or identified in the current version of the paper. The contribution would be strengthened by acknowledging this literature and clarifying which aspects remain missing in these studies (e.g., cross-country evidence or life-course perspective) and how the present analysis complements rather than replicates existing causal findings. See some of them:
o Persson, P., & Rossin-Slater, M. (2024). When dad can stay home: fathers' workplace flexibility and maternal health. American Economic Journal: Applied Economics, 16(4), 186-219.
o Bloom, N., Liang, J., Roberts, J., & Ying, Z. J. (2015). Does working from home work? Evidence from a Chinese experiment. The Quarterly journal of economics, 130(1), 165-218.
o Chuard, C. (2023). Negative effects of long parental leave on maternal health: Evidence from a substantial policy change in Austria. Journal of Health Economics, 88, 102726.

[RESPONSE] We thank this reviewer for bringing this important literature to our attention. In addition to our acknowledgement in the Introduction section (Lines 57-71), we now acknowledge in the Limitation section the challenge in producing causal estimates of work schedule arrangement on health and well-being due to its ethical concerns, but also acknowledge how other family- and/or work-friendly policies (e.g., work at home flexibility; Persson & Rossin-Slater, 2024; Bloom et al., 2015) holds great potential in contributing to the potential causal effects of work policies in addressing our health and well-being (Lines 677-688). As our focus of nonstandard work schedules may not align directly well with the implications for parental leave policy, we did not include the study by Chuard (2023), we hope you agree.

Other comments about writing
Introduction and motivation: The Introduction does not sufficiently clarify the motivation and contribution of the paper. It would benefit from a clearer articulation of the underlying theoretical mechanisms, a precise research question, and a concise explanation of how the paper answers this question and contributes to the existing literature. As currently written, the framing feels incomplete and makes it difficult to assess the paper’s value early on.

[RESPONSE] We thank this reviewer for this valuable suggestion. We now rephrase our Introduction to address these concerns, aiming to strengthen the paper's value early on (Lines 37-71).

Presentation of results: The results are largely presented as a sequence of estimates with little interpretation at the point of reporting. This gives the impression that the paper is primarily reporting numbers, with substantive discussion postponed to later sections. Integrating interpretation alongside the presentation of results is necessary to help readers understand the relevance and implications of the findings.

[RESPONSE] We thank this reviewer for this point, and we acknowledge that the differences in the content of the results section might have to do with preferences adopted by different fields. We are used to “reserving” interpretation for the Discussion section (and were told, in the past, by reviewers from other journals to do so). We now add interpretations right after describing some critical results for parsimony (Lines 462-477 and 548-566). We hope you agree with this middle ground.

We appreciate both reviewers very much for their very valuable and constructive comments. We believe the paper is now stronger because of these comments. Thank you.
